# A survey on ethical digital twins in healthcare

**DOI:** 10.3389/frai.2026.1875471

**Published:** 2026-09-21

**Authors:** Chaw Maung, Nia Richard, Shimei Pan

**Affiliations:** Information Systems Department, University of Maryland Baltimore County, Baltimore, MD, United States

**Keywords:** digital twin, healthcare, ethics, regulatory compliance, privacy and security, dynamic consent, ownership, trustworthy AI

## Abstract

Digital Twins for Health (DT4H), defined as dynamic, data-driven, *in silico* representations of individuals, organs, medical devices, clinical workflows, or healthcare systems, are emerging as a promising paradigm for transforming healthcare. DT4H systems have the potential to advance personalized medicine, treatment optimization, disease prediction, drug discovery, clinical trials, and health system planning. However, their real-world development and deployment also raise important ethical, legal, and social implications (ELSI). Because DT4H systems rely on sensitive longitudinal health data and may directly inform clinical decision-making, they introduce significant concerns related to their development, use, and ongoing governance. Many of these concerns are not entirely new, as they are grounded in established bioethical principles and substantially overlap with ELSI frameworks developed for AI for healthcare (AI4H). Accordingly, rather than examining DT4H-related ELSI issues in isolation, this survey situates them within existing bioethical and AI4H frameworks while identifying the distinctive challenges that arise from the unique characteristics of DT4H and must be addressed to support their ethical, responsible, and trustworthy development and deployment.

## 1 Introduction

Recent advances in digital twin (DT) technology allow us to create dynamic, individualized virtual representations of humans, organs, medical devices, clinical workflows, and healthcare systems. Although the concept of digital twins originated outside medicine, particularly in aerospace and manufacturing (Khan et al., 2025; Katsoulakis et al., 2024), recent advances in smart sensors, cloud computing, artificial intelligence, real-time simulation, and data analytics have accelerated the application of DT technology to complex human biological systems. Digital Twins for Health (DT4H) has the potential to move healthcare beyond a traditional reactive model toward a personalized, predictive, preventive, and participatory approach (Brand and Devlin, 2025). For example, a digital twin of the heart can simulate physiological processes, including blood circulation, cardiac rhythms, and organ-specific responses to different treatments, thereby supporting early heart disease prediction and personalized treatment planning. More broadly, DT4H systems can support disease prediction, personalized medicine, surgical optimization, drug discovery, clinical trial design, care coordination, and health system planning. By integrating heterogeneous, multimodal, multiscale patient data over time, these systems may significantly improve clinical processes, enhance decision-making, and improve quality of life (Ringeval et al., 2025; Jørgensen et al., 2023).

Despite their potential, DT4H systems may integrate complex mechanistic, statistical, and AI-driven models that raise significant ethical, legal, and social implications (ELSI). Their reliance on rich, sensitive, longitudinal, and highly personal data, combined with their potential influence on critical clinical decisions, creates important risks to patient safety, privacy, and autonomy. These concerns should therefore not be treated as a post-development checklist, but should be integrated throughout the design, development, deployment, and continuous monitoring of DT4H systems. Because a digital twin may function as a persistent digital surrogate of an individual, its technical reliability is closely intertwined with the ethical and governance framework that guides its development and use.

In this survey, we collected and analyzed recent literature on ELSI in DT4H systems. Rather than synthesizing existing ELSI themes in isolation, we proposed a multi-layered ELSI framework that situates ethical and responsible DT4H within the broader contexts of bioethics and ethical AI for healthcare (AI4H) systems. This framework illustrates the interconnections between ELSI issues in DT4H and AI4H while also highlighting DT4H-specific challenges, including continuous model updating and synchronization, simulation-based prediction, persistent digital representations of patients, and close coupling between physical individuals and their virtual counterparts. These distinctive features require existing ethical and governance frameworks to be further extended for the DT4H context.

The survey is organized as follows. We first provide background by defining DT4H and reviewing its major applications in clinical and healthcare domains, followed by a description of our survey methodology. We then summarize the main ELSI issues commonly discussed in the existing literature. Next, we propose a multi-layered ELSI framework for DT4H that is grounded in core bioethical principles, informed by existing AI4H frameworks, and extended to address DT4H-specific concerns. We further organize these ELSI considerations across key stages of the DT4H workflow, illustrating how they arise throughout the system lifecycle. Finally, we discuss key gaps and practical trade-offs and provide guidelines to support the responsible development and deployment of DT4H systems.

## 2 Background

### 2.1 Digital Twins for Healthcare (DT4H)

Digital twins are typically defined as living virtual models of physical entities or systems that continuously evolve in response to operational changes by incorporating real-time information and enabling prediction of the future states of their physical counterparts. In healthcare, a Digital Twin for Health (DT4H) system can be broadly defined as a living, data-driven, *in silico* replica of a health-related entity or process that is continuously updated with multimodal and multiscale data to support dynamic simulation of potential treatment strategies, monitoring and prediction of health trajectories, and early intervention and prevention (Katsoulakis et al., 2024). Typical data used in DT4H may include Electronic Health Records (EHRs), biometric data, wearable sensor signals, imaging data, multi-omics data such as genomic and proteomic data, environmental data, and social factors (Katsoulakis et al., 2024).

As shown in Figure 1, a typical DT architecture consists of three main components: a physical entity, such as a person; a virtual replica of that entity; and a real-time, bidirectional data connection that synchronizes the physical and digital counterparts. A DT is not a standalone technology but rather an integrated system that combines multiple enabling technologies (Popa et al., 2021). The virtual model simulates the behavior of the physical entity using computational, mechanistic, and AI/ML modeling techniques, while technologies such as the Internet of Things (IoT), cloud computing, and large-scale biomedical data infrastructures support continuous data collection, updating, and adaptation between the physical and virtual entities.

**Figure 1:**
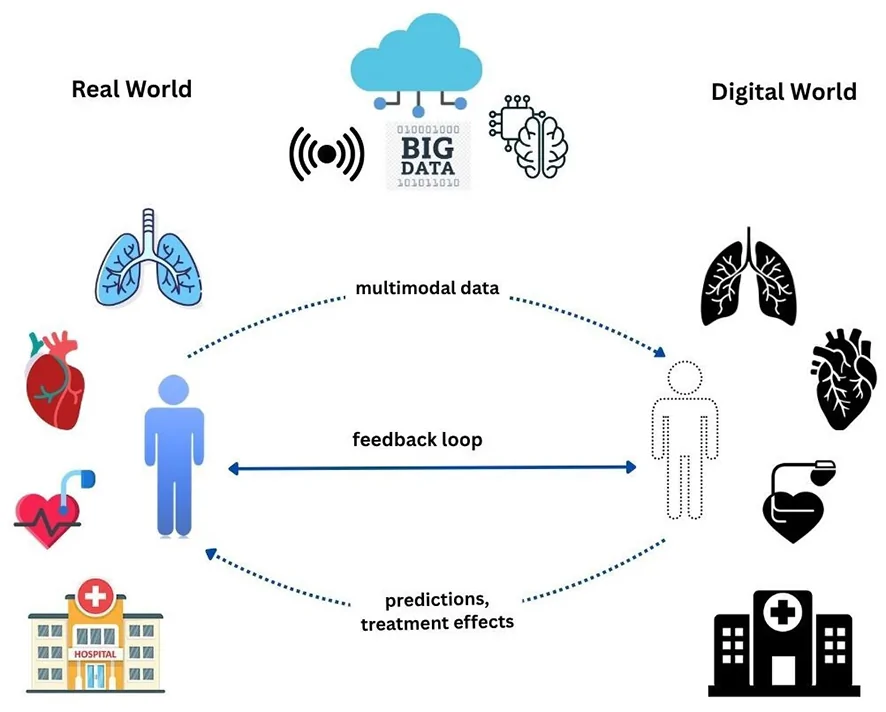
Conceptual overview of DT4H framework.

Compared with typical AI4H systems, DT4H systems exhibit several distinctive characteristics. First, a DT4H system is often built around an explicit digital representation of a physical entity, whereas most AI4H systems do not explicitly create such a representation. Moreover, the virtual representation is not static; rather, it is a living model continuously and dynamically updated as new data streams become available. Additionally, DT4H systems can enable what-if simulation, or counterfactual reasoning, to support healthcare professionals with decision-making options that go beyond prediction alone.

At the same time, DT4H systems share important attributes with AI4H systems. Both are data-driven systems that may inform critical, and sometimes life-or-death, healthcare decisions with significant social and economic consequences for large populations. Both also rely on sensitive healthcare data. In addition, significant uncertainty exists in their modeling and prediction processes as they often depend on how the data are noisy, incomplete, or biased.

According to Khoshfekr Rudsari et al. (2025), typical DT4H systems can be classified into four types. Component twins represent anatomical substructures or specific functions, such as coronary vessels. Asset twins are replicas of whole human organs or physical assets, such as the heart, brain, or medical devices. System twins capture complex interactions among multiple components within a broader system, such as the human body or specific physiological systems, including the circulatory system. Process twins represent larger healthcare infrastructure and operational workflows, supporting simulation and optimization of healthcare delivery processes (Ringeval et al., 2025).

### 2.2 Typical DT4H applications

**Personalized Precision Medicine:** Personalized precision medicine enables patient-specific simulations that tailor medical interventions to individual patients rather than relying on standardized care. It is one of the most common applications of DT4H. An individual digital twin is developed by integrating genomic, physiological, environmental, and lifestyle data with the patient's clinical records, including laboratory results, medical imaging, and real-time inputs from wearable devices or IoT sensors (Venkatesh et al., 2024). DT4H has been used in managing chronic diseases such as diabetes, cardiovascular diseases, oncological diseases (Elgammal et al., 2025), and rare pediatric diseases, e.g., Phenylketonuria (PKU) (März et al., 2025). Moreover, they can predict disease progression, enabling early intervention to improve patient wellbeing and health outcomes (Katsoulakis et al., 2024).

**Surgical Planning and Organ Transplant:** DTs can create digital organ or disease models that allow surgeons to rehearse complex procedures, optimize prosthetic device fitting (Elgammal et al., 2025), and evaluate alternative treatment strategies, particularly for high-stakes complex neurosurgical or oncological procedures (Ringeval et al., 2025). Similarly, DTs can support time-sensitive organ transplantation by generating virtual replicas of donor and recipient organs for quality assessment, preoperative functional simulation, and continuous post-operative monitoring (Xames, 2025).

**Clinical Trials and Drug Discovery:** New medicine development is traditionally a time-consuming, labor-intensive, costly, and high-risk process, often taking an average of 12–15 years to reach the market and requiring clinical trials involving thousands of patients (Sun and Ran, 2025). DT4H systems can address these challenges by enabling *in silico* clinical trials with diverse virtual patient populations constructed from multi-institutional data. These models allow researchers to evaluate the efficacy and safety of candidate treatments and medical devices, as well as cohort-level responses, while reducing the need for human subjects, shortening development timelines, and lowering risks to patients (Khoshfekr Rudsari et al., 2025).

**Hospital and Workflow Management:** DTs simulate virtual replicas of hospital infrastructure and operational environments, enabling administrators to monitor healthcare workflows in real time, including operating room availability, ventilator supply, hospital bed occupancy, staff workload, and patient flow. These models support more efficient clinical workflows, optimized resource management, and improved staff scheduling (Zhang et al., 2024; Venkatesh et al., 2024). The value of this approach was demonstrated during the COVID-19 pandemic, when hospitals used simulation models to improve operational workflows and supply chain management, reduce patient waiting times, and maintain quality of care during periods of peak demand (Menon et al., 2025).

Although DT4H technology has significant potential to simulate disease processes, advance drug discovery, and tailor medical care, it also carries substantial risks. In the following section, we critically analyze the existing literature on ELSI in DT4H to assess the current state of the field, identify key gaps and limitations, provide practical guidelines, and suggest future research directions.

## 3 Survey methodology

Because ELSI in DT4H is an emerging and interdisciplinary area, we conducted a scoping review guided by established scoping-review methodology, following the PRISMA-ScR (Tricco et al., 2018; Peters et al., 2020) framework to support transparent and reproducible reporting of our scoping review. The goal was to map the existing literature, identify major themes and gaps, and inform future research directions.

**Search Sources and Queries:** We searched five main databases for research publications: *Google Scholar, Web of Science, PubMed, IEEE Xplore*, and the *ACM Digital Library*. These sources were selected to provide broad coverage of biomedical, healthcare, engineering, and computer science literature relevant to DT4H and its ELSI dimensions. Scopus and Embase were not included because access to these subscription-based databases was not available through our institutional resources.

Search queries were designed and iteratively refined to capture the most relevant and recent literature on this topic. The query design was guided by three criteria. First, we used the exact keywords “digital twin” or “digital twins” to retrieve publications related to digital twin technology. Second, we included terms such as “health,” “healthcare,” “medicine,” and “biomedical” to further narrow down the search to healthcare-related applications. Third, we incorporated ELSI-related keywords, including “ethical,” “legal,” “law,” “regulation,” “governance,” “societal,” and “socio-ethical,” to identify papers addressing ethical, legal, and social implications. Because DT4H is an emerging area, we restricted the search period to publications from 2018 to March 2026, when the list of publications included in this survey was last updated. We focused the search on article titles and abstracts to ensure that the retrieved publications were primarily concerned with DT4H and its ELSI dimensions. As shown in Table 1, we applied the same conceptual query adapted to each platform's query syntax.

**Table 1:** Search queries used in retrieving publications from five databases.

Exact search strings used in paper retrieval	Database	Results
(TI=(“Digital Twin*”) AND TI=(Healthcare OR Health OR Medicine OR Biomedical) AND AB=(ethical OR legal OR law OR governance OR “socio-ethic” OR societal OR regulation OR regulatory))	Web of Science	76
allintitle: (“Digital Twin” OR “Digital Twins”) (healthcare OR health OR medicine OR biomedical) (ethical OR legal OR law OR governance OR “socio-ethic” OR societal OR regulation OR regulatory)	Google Scholar	28
(((“digital twin”[TITLE] OR “digital twins”[TITLE]) AND ((healthcare[TITLE]) OR (health[TITLE]) OR (medicine[TITLE]) OR (medical[TITLE]) OR (biomedical[TITLE])) AND ((ethical[TITLE/ABSTRACT]) OR (legal[TITLE/ABSTRACT]) OR (law[TITLE/ABSTRACT]) OR (governance[TITLE/ABSTRACT]) OR (regulation[TITLE/ABSTRACT]) OR (regulatory[TITLE/ABSTRACT]) OR (“socio-ethic”[TITLE/ABSTRACT]) OR (societal[TITLE/ABSTRACT]))))	PubMed	41
((“Document Title”:“Digital Twin” OR “Document Title”:“Digital Twins”) AND (“Document Title”: Healthcare OR “Document Title”:Health OR “Document Title”: “Medic*” OR “Document Title”:“Biomedical”) AND (ELSI OR ethic* OR legal OR law OR Governance OR social OR societal OR “socio-ethic*” OR regulation* OR regulatory OR privacy))	IEEE Xplore	79
Title:((Digital Twin OR Digital Twins)) AND Title:((Healthcare OR Health OR Medicine OR Biomedical) OR Abstract:((Ethics OR Ethical OR Legal OR law OR regulation OR Governance OR policy OR socio-ethical OR socio-ethics OR societal OR Bioethical))	ACM	19

The search included English-language publications, such as journal articles, conference papers, literature reviews, and case studies. Except for academically defended doctoral dissertations, we only included peer-reviewed publications. Duplicate records were excluded. In total, 243 articles were retrieved. After 41 duplicate records, 3 non-English papers, and 10 inaccessible records were removed, there were 189 records for eligibility screening. We then manually screened these documents to verify whether each article substantively discusses at least one ELSI concern related to DT4H. The main exclusion criteria used are summarized in Table 2. The screening process is broken down using a PRISMA-ScR flow diagram (shown in Figure 2) which includes the number of records identified, duplicates and inaccessible records removed, records screened, records excluded, and studies included in the final synthesis.

**Table 2:** Exclusion criteria.

Exclusion criteria	Description
Irrelevant content for DT4H	Papers were excluded if they focused on digital twin frameworks, architectures, computational methods, applications, or technical challenges but did not address ELSI-related issues.
Insufficient ELSI content	Papers were excluded if they discussed DT applications in healthcare and briefly mentioned ELSI issues, but ELSI discussions were not substantive.
Full text unavailable	Papers were excluded if the full text was inaccessible.
Non-English publication	Publications not available in English were excluded because the review was limited to English-language literature.
Outside the publication period	Publications outside the 2018–March 2026 date range.
Non-peer reviewed publications	Publications that had not undergone peer review, such as preprints on arXiv, were excluded; however, doctoral dissertations were included because they typically undergo rigorous academic review and evaluation.

**Figure 2:**
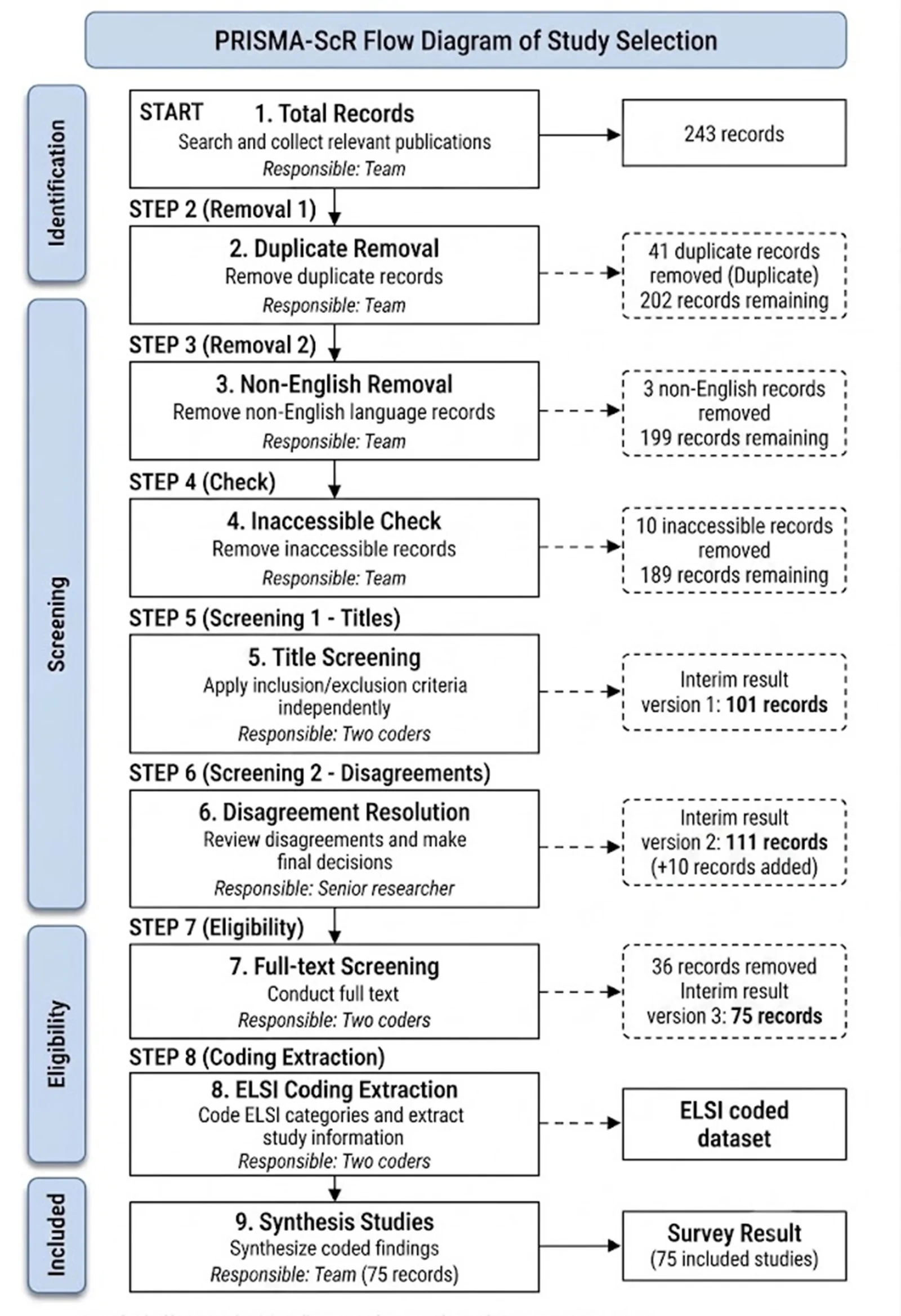
PRISMA flow diagram.

**Content Screening Method:** Two independent human coders manually screened the titles and abstracts of all 189 records for inclusion. The coders agreed on 87.8% of the screening decisions, with Cohen's kappa of κ = 0.764, indicating substantial agreement beyond chance. Based on this initial screening, 101 papers were included by agreement of both coders. For the 23 cases with inter-coder disagreement, a senior researcher specializing in ethical digital twins adjudicated the conflicts and selected 10 additional papers for further consideration. To further assess the relevance of the resulting 111 papers, the two coders conducted full-text screening, yielding a final dataset of 75 papers.

**ELSI Theme Coding Method:** We followed a procedure similar to that used in the content-screening stage to assign one or more ELSI theme labels to each paper included in the survey. The coders selected the applicable labels from a predefined set of principal ELSI categories. Figure 3 shows the Top-10 most frequently occurring ELSI themes discussed in the surveyed papers.

**Figure 3:**
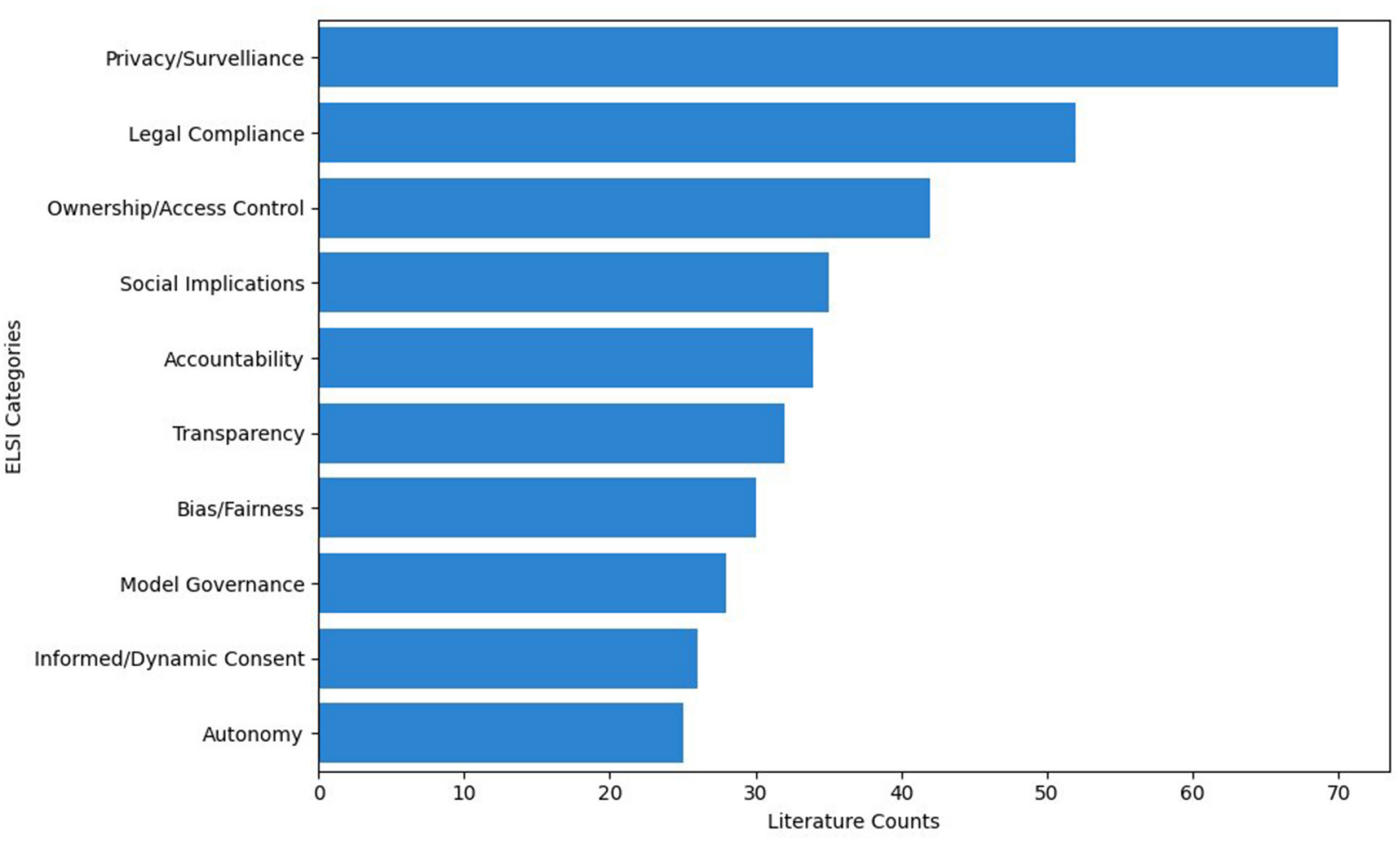
Frequency of ELSI categories discussed in reviewed literature.

## 4 Analyzing and synthesizing the main ELSI themes

### 4.1 Summary of the main themes in survey papers

Across the 75 studies, ethical, legal, and social implications of DT4H were frequently discussed as interconnected. This interconnectedness adds a layer of systemic complexity to the digital health ecosystem, highlighting the need to address these dimensions as an integrated whole rather than as separate challenges. Our survey also reveals a clear imbalance in the attention given to different ELSI challenges in DT4H. As shown in Figure 3, *data privacy and security* dominate the discussion, appearing in 70 of the 75 studies. These discussions primarily address concerns about the extensive use of individual health data in DT4H systems. *Legal compliance* is the second most frequently discussed category, identified in 52 studies and underscoring the importance of regulatory requirements and data-governance policies in DT4H innovation. *Ownership and access control* ranks third, appearing in 42 studies, with considerable attention given to who should own digital twins and who should be permitted to access and control their data, including after a patient's death. *Social implications* are addressed in 35 studies, examining how DT4H may reshape traditional treatment settings through increased reliance on digital simulations. Other frequently discussed concerns include *accountability* (34 studies), *transparency* (32 studies), and *bias and fairness* (30 studies), highlighting the need for responsible decision-making, explainable, and equitable DT4H systems. *Model governance* is discussed in 28 studies, focusing on the importance of model fidelity and reliability in continuously evolving DT4H systems, while *dynamic consent* appears in 26 studies, addressing the limitations of one-time fixed consent and the need for ongoing consent mechanisms for real-time data collection in DT4H systems. Finally, *autonomy* is discussed in 25 studies, emphasizing the importance of preserving human oversight within the decision loop and maintaining control over machine-driven decisions. More details on how these topics are covered in the surveyed papers can be found in Table 3. In the following, we discuss each of these major concerns in greater detail.

**Table 3:** Analysis of ELSI issues in DT4H literature.

References	Privacy/Surveillance	Dynamic Consent	Trans- parency	Account-ability	Bias/Fairness	Autonomy	Model Governance	Ownership/ Access Control	Legal Compliance	Social Implications
Story (2023)	✓							✓	✓	✓
Menon et al. (2025)	✓		✓	✓	✓				✓	
Iqbal et al. (2022)	✓			✓		✓		✓	✓	✓
Zoltick and Maisel (2023), Vasiliu-Feltes et al. (2023), Samei (2025)	✓	✓								
Lal et al. (2022), Borrelli et al. (2025), Huang et al. (2022), Barbier et al. (2025)	✓	✓	✓	✓	✓	✓	✓		✓	
Iqbal et al. (2025)	✓	✓	✓			✓	✓			✓
Milosevic and Van Schalkwyk (2023)	✓		✓	✓	✓	✓				✓
Sun and Ran (2025)	✓	✓	✓	✓	✓		✓	✓		
Delerm and Pilottin (2024)	✓				✓					✓
Begishev et al. (2024), Tahir et al. (2026), Xu et al. (2026)	✓			✓		✓				✓
Jørgensen et al. (2023)	✓			✓						
Kuersten (2023), Sharma et al. (2024), De Maeyer and Renzi (2026)	✓	✓		✓					✓	
Burr et al. (2026)	✓		✓	✓	✓		✓	✓	✓	✓
Shao and Ou (2025)	✓	✓		✓	✓			✓		✓
März et al. (2025)		✓	✓	✓				✓	✓	✓
Popa et al. (2021)	✓			✓		✓		✓		✓
Teller (2021), Pandurangan et al. (2025), Ibrahim and Salhab (2023)	✓		✓	✓				✓	✓	
Boretti (2025), Pisirgen and Hiziroglu (2025)	✓		✓	✓			✓	✓	✓	✓
Vallée (2026), Alimour and Alrabeei (2024)	✓	✓			✓					✓
Brand and Devlin (2025)	✓	✓		✓		✓			✓	
Braun (2022)		✓	✓		✓			✓		✓
Tretter (2021)				✓				✓		✓
Jabin et al. (2024)	✓		✓	✓		✓		✓	✓	✓
Venkatesh et al. (2024), Vithya and Valarmathie (2024)	✓				✓				✓	✓
Amram et al. (2023)	✓	✓						✓	✓	
De Kerckhove (2021), Braun and Krutzinna (2022)	✓					✓		✓		
Bruynseels et al. (2018), Drummond and Coulet (2022)	✓		✓			✓				✓
Guerrero Quiñones and Puzio (2025)					✓	✓				✓
Kamel Boulos and Zhang (2021)	✓							✓		✓
Zhang et al. (2024), Olawade et al. (2026), Pan et al. (2025)	✓	✓	✓		✓			✓	✓	
Coorey et al. (2022)	✓				✓			✓	✓	✓
Xames (2025)	✓							✓	✓	
Ringeval et al. (2025)	✓		✓				✓	✓		✓
Kumar et al. (2025)	✓	✓	✓		✓	✓	✓		✓	
Acevedo et al. (2026)	✓		✓		✓				✓	✓
Iliuṭă et al. (2025)	✓	✓	✓			✓				✓
Venkatraman et al. (2026), Attaran and Attaran (2024)	✓			✓			✓	✓	✓	
Lomotey et al. (2024)	✓			✓		✓	✓	✓		
Mishra et al. (2025)	✓						✓		✓	✓
Judijanto (2026)	✓						✓		✓	
Deka et al. (2026)	✓		✓		✓			✓	✓	
Narigina et al. (2024)	✓			✓			✓	✓	✓	✓
Leong (2025)	✓		✓	✓		✓	✓	✓	✓	✓
Silva and Vale (2025), Li et al. (2025)	✓			✓	✓		✓	✓	✓	✓
Armeni et al. (2022), Vanin et al. (2026)	✓						✓	✓	✓	
Vasiliu-Feltes et al. (2025)	✓	✓	✓	✓				✓	✓	✓
Verhees et al. (2026), Antoun et al. (2026), Khoshfekr Rudsari et al. (2025)	✓				✓		✓	✓	✓	
Piechowiak et al. (2026)					✓				✓	
Carbonaro et al. (2025)	✓		✓		✓	✓	✓		✓	✓
Saratkar et al. (2025)	✓					✓	✓	✓	✓	

Data privacy and security are the most discussed issues, underpinning many of the other concerns identified in this review. A breach or manipulation of data in digital twins can directly jeopardize patient autonomy, trust in the patient-clinician relationship, and patient safety, and expose sensitive personal information while also raising broader social justice issues. Because DT4H systems rely on large volumes of personal health information (PHI), biometric data, and other sensitive longitudinal data, unauthorized access or exposure can create serious and potentially irreversible risks, including risks associated with the misuse or manipulation of genetic information (Story, 2023; Iqbal et al., 2022). For example, if a patient's predicted risk of future chronic disease is disclosed to third parties or accessed by insurers, it could result in denial of coverage if such predictions are treated as pre-existing conditions (Abrahams, 2025). Data breaches may also disproportionately harm marginalized populations. For transgender individuals, for instance, exposure of sensitive identity-related information could lead to severe socio-ethical harms, including gender discrimination and barriers to healthcare and housing (Boretti, 2025).

Cybersecurity risks are particularly acute in DT4H systems because malicious actors may interfere not only with data but also with medical decisions and indirectly affect human biological systems through live data streams transmitted by IoT devices (Menon et al., 2025). In addition, continuous monitoring and extensive data collection raise concerns about surveillance, threats to personal autonomy, and intrusion into family privacy (Drummond and Coulet, 2022). These privacy and surveillance concerns are directly related to patient autonomy, which must be preserved throughout the DT lifecycle. Fundamentally, patients have the right to make health-related decisions based on their values, beliefs, and preferences, rather than relying solely on automated recommendations or decisions (Sun and Ran, 2025). Autonomy issues may be particularly consequential for vulnerable populations who have limited ability to understand, control, or mitigate potential harms. For instance, children and some individuals with disabilities may require parents or legal guardians to provide informed consent. Although proxy authorization can enable participation when individuals cannot independently provide consent, it can also complicate family decision-making and potentially diminish individual empowerment (Teller, 2021; Olawade et al., 2026).

Moreover, these implications contribute to a deeper structural problem: the insufficiency of one-time fixed consent in evolving DT4H environments. Dynamic consent was examined as a potential mechanism for addressing this limitation by allowing individuals to modify their consent preferences over time. For instance, a critical question here is whether the consent given by parents remains valid once the child reaches adulthood, or whether a clear voluntary opt-out process exists (März et al., 2025). The surveyed literature also highlights key obstacles to implementing dynamic consent in DT4H systems, including user exhaustion caused by excessive consent requests and difficulties obtaining approval from unconscious patients, such as those who are in comas. Additional concerns arise when patients withdraw consent over time during continuous DT feedback loops, as this may adversely affect clinical decisions and outcomes (Zhang et al., 2024).

Beyond concerns about how data are collected and controlled, the characteristics and representativeness of those data also shape the bias and fairness challenges of DT4H systems, with particular attention to the demographic under-representation or the unrepresentative training datasets. This issue is not only a socio-ethical concern but also a functional limitation of DT4H systems. AI-based predictive models may learn biological patterns that reflect the demographic groups historically overrepresented. For example, datasets historically derived from middle-aged men of European descent may produce inaccurate predictions and inadequate healthcare recommendations for young Asian women. Such bias can perpetuate existing health inequities and distort clinical understanding. Moreover, DT4H systems often require pristine, standardized datasets, whereas real-world clinical data are typically messy, fragmented, incomplete, and distributed across different institutions.

Ensuring DT4H models use representative and reliable data is not enough for responsible clinical use. Models must also be explainable for clinicians and patients to understand how predictions and recommendations are generated. This need for explainability is closely connected to accountability because it is crucial to determine who is accountable among various stakeholders when DT-generated decisions result in errors or harm, particularly when responsibility is distributed among multiple stakeholders throughout the DT lifecycle. Accordingly, transparency, accountability, ownership, and access control are frequently discussed together in the DT4H literature.

Transparency is another recurring concern, particularly because complex AI models may be difficult for both patients and clinicians to understand. This includes questions about how data are used, how models operate, and how decisions or recommendations are generated (Milosevic and Van Schalkwyk, 2023). In high-stakes contexts such as disease diagnosis, treatment planning, and healthcare administration, transparency is a key requirement for both accountability and informed consent. Without sufficient transparency, it becomes difficult to assign responsibility, determine liability, and obtain meaningful consent across the DT4H lifecycle (Borrelli et al., 2025). These accountability challenges are further complicated by questions of ownership and access control, particularly as the increasing capabilities of AI systems blur the boundaries between human- and machine-generated outputs. In DT4H, this raises questions regarding digital property rights, permissions to control digital simulations, governance of DT data and models, and the status of digital assets after a person's death, especially when multiple stakeholders are involved.

Building on these interconnected ethical concerns, the literature also examines the legal and regulatory frameworks governing DT4H systems, particularly the extent to which existing frameworks adequately address challenges related to data security and privacy, model reliability, accountability, ownership, and access control. Regulatory compliance is discussed across 52 studies, with particular attention to the limitations of applying existing legal frameworks to DT4H. For example, the General Data Protection Regulation (GDPR) governs the processing of personal data and establishes requirements concerning lawful processing, transparency, data-subject rights, and, where applicable, consent, while the Health Insurance Portability and Accountability Act (HIPAA) protects identifiable health information within covered healthcare contexts (Vithya and Valarmathie, 2024). However, these frameworks were not developed specifically for dynamic, continuously evolving, and highly integrated DT environments, creating challenges when they are applied to multidimensional phenotypic, clinical, and other longitudinal health data. Similarly, the Genetic Information Non-discrimination Act (GINA) provides protections against specified uses of genetic information, particularly in employment and health insurance contexts; however, its protections may not fully encompass the broader predictive biological profiles and derived health information generated by DT4H systems (Story, 2023). Regulatory guidance for Software as a Medical Device (SaMD) also continues to evolve as AI/ML-driven diagnostic and clinical technologies become more prevalent, including guidance from the Food and Drug Administration (FDA) in the United States and the European Union Medical Device Regulation (MDR) (Lal et al., 2022). In addition, existing intellectual property laws face challenges in determining ownership and legal protection for machine-generated algorithms and other computational outputs (Zoltick and Maisel, 2023). Collectively, the literature highlights the need for adaptive, comprehensive, and cross-jurisdictional legal and regulatory frameworks that can accommodate the data-intensive, dynamic, and continuously evolving nature of DT4H systems.

While regulatory frameworks provide mechanisms for addressing these technical and ethical challenges, these implications go beyond individual rights and institutional responsibilities to broader societal outcomes. Broader societal implications are examined alongside their conceptually intertwined ethical risks. The existing literature primarily focuses on health disparities among marginalized groups, including low-income families, older adults, and queer communities (Jabin et al., 2024; Guerrero Quiñones and Puzio, 2025). Furthermore, in modern clinical settings, the heavy reliance on AI-driven personalized medicine may jeopardize the patient-clinician relationship when doctors engage with simulating and analyzing patients' digital twins instead of interacting with and treating real human patients. It could reshape traditional clinical practices (Iqbal et al., 2022), and lead to potential loss of trust, empathy, and interpersonal communication (Popa et al., 2021). Moreover, precision medicine, supported by advanced simulation, can extend normal human capabilities beyond therapeutic purposes. For example, medications used to help patients with ADHD improve attention and focus may also be used to enhance cognitive performance in healthy individuals. This illustrates the blurred boundary between treatment and enhancement (Ferlito et al., 2024).

Finally, these interconnected ELSI concerns highlight the importance of governing DT models throughout their lifecycle. DT model performance depends heavily on data availability and quality. Minority populations, people with disabilities, children with rare pediatric diseases, and queer communities are often underrepresented in available datasets. Such data imbalances can perpetuate existing health disparities and underscore the need for robust model governance to mitigate those risks throughout the DT lifecycle.

### 4.2 Proposed multi-layered contextual framework for ELSI in DT4H

While the previous section examined the primary ELSI themes in DT4H, it is important to situate this analysis within the broader and more established contexts of bioethics and ELSI in AI for healthcare (AI4H). Without this broader perspective, the discussion risks becoming fragmented and overlooking important connections among related ethical, legal, and social concerns. Linking ELSI in DT4H with foundational bioethical principles and AI4H ELSI frameworks helps identify “inherited” ethical challenges that may be underexamined in current DT4H discourse. At the same time, comparison with AI4H also clarifies where DT4H requires additional extensions and specialized considerations. Recognizing both the shared foundations and the distinct technical characteristics of DT4H can support the development of a more robust ethics-by-design framework, ensuring that these systems are not only technologically advanced but also socially responsible, clinically trustworthy, and legally sound.

To ground our analysis in the rich literature on bioethics and ethical AI4H, we introduce a multi-layered ELSI framework for DT4H. As shown in Figure 4, this framework begins with core bioethical principles, examines how they are instantiated in AI4H systems, and then further specializes these concerns within the DT4H context. These three layers are interdependent: bioethical principles guide the development of AI4H ELSI frameworks, which are then extended to address DT4H-specific risks. This layered structure provides a coherent way to categorize ELSI issues in DT4H and offers insight into how ethical risks may emerge and interact.

**Figure 4:**
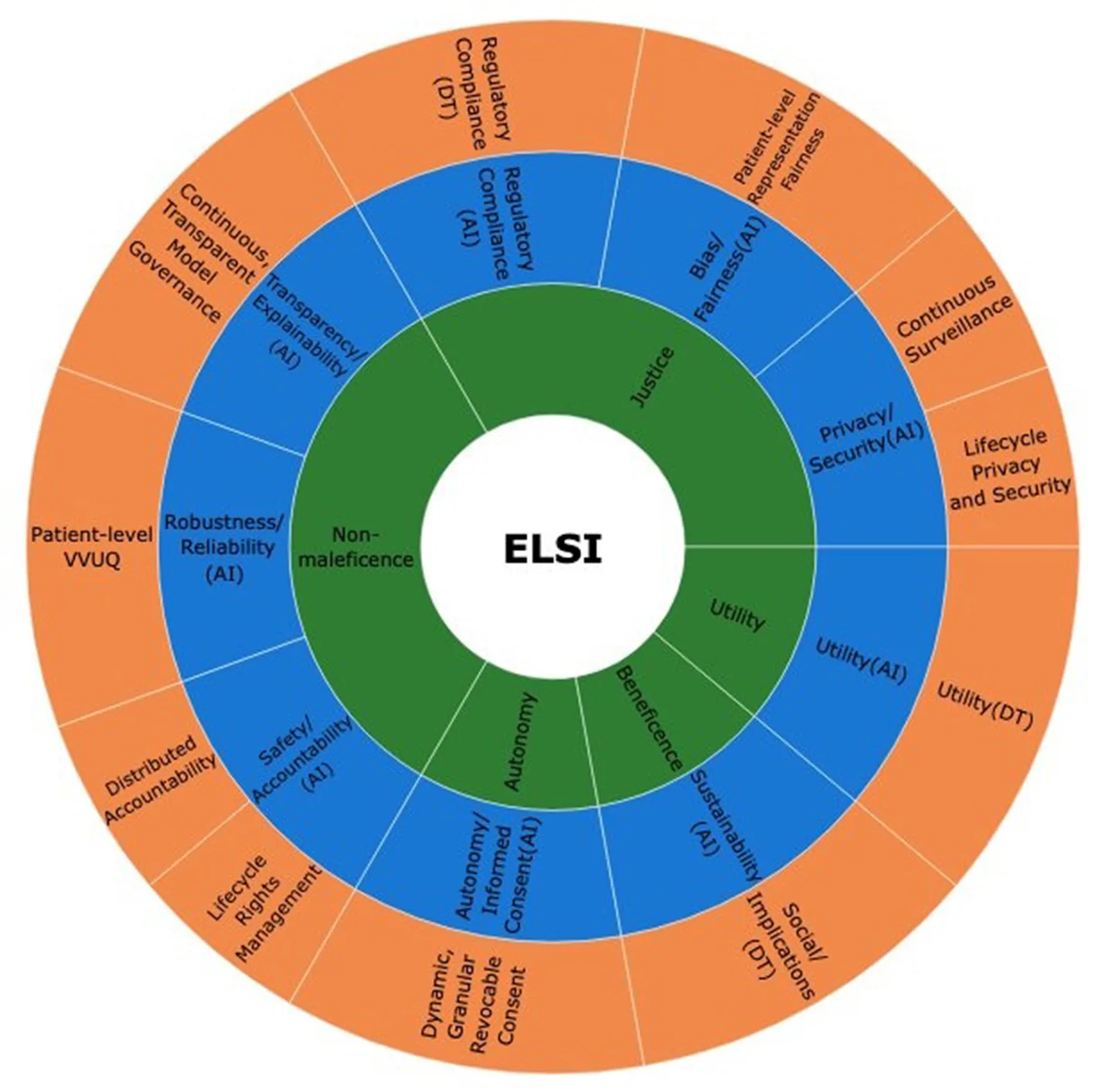
Multi-layer structure of ELSI framework in DT4H.

#### 4.2.1 Core bioethical principles

General bioethical principles used to guide human-subjects research and medical interventions include respect for autonomy, beneficence, non-maleficence, and justice.

**Autonomy:** Individuals have the right to self-determination with respect to their treatment plans and clinical interventions. This includes a patient's decision to provide informed consent for the collection, storage, and use of their personal health data with a full understanding of how the data and models trained on them influence clinical decision-making and treatment recommendations. Informed consent is a prerequisite of autonomy (Varkey, 2021).

**Justice:** Individuals are entitled to equitable access to clinical care and the fair distribution of medical resources and digital technologies (Busch et al., 2023). Achieving this requires actively addressing disparities in data representation, healthcare infrastructure, and digital literacy, as failure to do so may reinforce existing systemic health inequities.

**Non-maleficence:** The obligation to avoid or minimize harm. It is often summarized as “do no harm.” For medical interventions and human-subjects research, this principle requires risk minimization, safety monitoring, clinical validation, protection from unnecessary procedures, and stopping or modifying a study when risks become excessive.

**Beneficence:** This requires researchers and clinicians to promote the welfare of patients or participants. In practice, this means designing studies or interventions that have a reasonable prospect of benefit, scientific value, and clinical relevance.

Additionally, some bio-ethics frameworks also include an additional principle on Utility. This principle is closely related to the Non-maleficence and Beneficence principles. According to Lal et al. (2022), the principle of *Utility* emphasizes maximizing the benefits while minimizing the harm to the vast majority of the population.

In practice, these principles are reflected in foundational biomedical ethics frameworks such as the Belmont Report (Beauchamp, 2008), the Declaration of Helsinki (World Medical Association, 2013), and the CIOMS International Ethical Guidelines for Health-related Research Involving Humans (Council for International Organizations of Medical Sciences, 2016).

#### 4.2.2 ELSI frameworks for AI4H

As AI becomes increasingly integrated into biomedical research and healthcare, AI4H systems must be developed and deployed in ways that protect patients, support clinicians, and promote responsible use. General bioethical principles provide a normative foundation for AI4H by translating broad moral obligations into practical requirements for healthcare AI. For example, autonomy motivates transparency, explainability, informed consent, and meaningful human oversight; beneficence supports clinical effectiveness and improved health outcomes; non-maleficence underpins safety, robustness, privacy, and protection against system failure or misuse; justice motivates fairness, non-discrimination, and equitable access; and utility emphasizes maximizing overall benefit while minimizing cost and systemic risk.

These principles are reflected in major international AI ethics and governance frameworks, including guidance from the World Health Organization (WHO), the Organization for Economic Co-operation and Development (OECD), and the European Commission, as well as operational frameworks such as the NIST AI Risk Management Framework (AI RMF) and the FUTURE-AI guideline (World Health Organization, 2021; OECD, 2024; National Institute of Standards and Technology, 2023; European Commission, 2019; Lekadir et al., 2025). For example, WHO guidance emphasizes human autonomy, wellbeing and safety, transparency, responsibility, inclusiveness, equity, and sustainability, while FUTURE-AI organizes trustworthy healthcare AI around fairness, universality, traceability, usability, robustness, and explainability (World Health Organization, 2021; Lekadir et al., 2025).

Across these frameworks and the broader AI4H literature, several recurring ELSI concerns emerge. These include *privacy and security*, particularly because healthcare AI relies on large volumes of sensitive and distributed health data (Mbiazi et al., 2025; Karimian et al., 2022); *bias and fairness*, as unrepresentative datasets may produce unequal performance and reinforce existing health disparities (Mbiazi et al., 2025; Masciari et al., 2024); *transparency and explainability*, which are necessary for clinicians and patients to understand and appropriately evaluate AI-supported decisions (Mbiazi et al., 2025); and *autonomy and informed consent*, which require patients to retain meaningful control over their data and healthcare decisions (Karimian et al., 2022). AI4H frameworks also emphasize *robustness, reliability, safety, and accountability*, particularly because errors in high-stakes clinical systems can result in patient harm and responsibility may be distributed across clinicians, developers, healthcare institutions, and technology providers (Karimian et al., 2022; Kiseleva et al., 2022). Finally, regulatory compliance, sustainability, and broader social implications, including potential over-reliance on automated decision-making and effects on patient-clinician relationships, remain important concerns in responsible AI4H (Qian et al., 2024; Mbiazi et al., 2025; Karimian et al., 2022).

Together, these established AI4H principles provide the baseline for understanding ELSI in DT4H. However, as discussed in the following section, the persistent, continuously evolving, patient-specific, and simulation-based characteristics of digital twins introduce additional challenges that require these principles to be further specialized and extended.

#### 4.2.3 Extensions and specializations for DT4H

In this section, we examine DT4H-specific ELSI extensions that build on, but extend beyond, established ethical and governance frameworks for AI4H. DT4H and AI4H systems share many fundamental characteristics. Consequently, DT4H inherits many of the core ELSI concerns already recognized in AI4H. However, DT4H also introduces distinctive characteristics, which create additional risks and governance challenges that are not fully captured by existing AI4H frameworks, motivating the need for DT4H-specific extensions.

**Data Privacy, Security and Re-identification Risks:** Privacy and security concerns in DT4H extend beyond those typically encountered in conventional AI4H because a medical digital twin depends on extensive, longitudinal, and highly sensitive data that may include genomic information, EHRs, medical imaging, biometric signals, continuous wearable-sensor streams, behavioral patterns, and everyday-life data. Unlike many conventional AI4H systems that operate on episodic snapshots of patient data, DT4H systems are designed to maintain an evolving representation of a specific individual through continuous and often bidirectional data flows. This persistence substantially increases the volume, granularity, and duration of data exposure and therefore increases the potential risks and consequences of data breach and unauthorized access (Jørgensen et al., 2023; Huang et al., 2022; Bruynseels et al., 2018). For example, compromising a conventional diagnostic model may expose a single imaging study or prediction, whereas compromising a patient's digital twin could reveal years of clinical history together with real-time physiological measurements, genomic characteristics, inferred disease risks, and simulated future health trajectories. DT4H systems also generate large amounts of derived, synthetic, and counterfactual information that may never have been directly recorded in the medical record, such as an inferred high probability of a future neurodegenerative condition. Such information creates additional questions about access, disclosure, and protection because it may be highly consequential even though it is computationally inferred rather than clinically observed. Moreover, the richness and longitudinal integration of these data also heighten re-identification risk. Even when direct identifiers are removed, combinations of genomic attributes, physiological traces, mobility or behavioral patterns, and wearable-device data can create highly distinctive individual profiles, making conventional one-time de-identification increasingly fragile. Because DT4H systems may function as persistent digital surrogates of patients, privacy and security should therefore be treated as lifecycle governance requirements, encompassing data collection, integration, updating, inference, simulation, de-identification, sharing and eventual archival or deletion, rather than as isolated data-protection measures applied only at the point of collection.

**Continuous Surveillance and Autonomy:** DT4H systems can substantially intensify surveillance-related concerns already present in AI4H because high-fidelity digital twins require sustained, multimodal, and multiscale observation of individuals over time. Unlike many conventional AI4H systems that rely on discrete clinical encounters or episodic data snapshots, a digital twin may integrate data from comprehensive sources continuously to remain synchronized with its physical counterpart. Huang et al. (2022) describe this extensive data acquisition as *hypercollection*, noting that digital twins may incorporate not only clinical information but also behavioral, social, occupational, and other contextual data. In this way, routine aspects of daily life can become ongoing sources of health-related surveillance.

The ethical significance of this surveillance extends beyond the amount of data collected. By integrating multiple data streams over time, DT4H systems may infer sensitive characteristics that were never explicitly disclosed or directly measured. For example, changes in sleep, mobility, heart rate, and smartphone activity might collectively reveal deteriorating mental health, pregnancy, substance use, or the early onset of dementia. Huang et al. (2022) specifically identify risks from inference based on the aggregation of diverse data sources, while Bruynseels et al. (2018) argue that combining biological and lifestyle information can reveal substantially more about an individual than isolated biomedical datasets. As the twin becomes more comprehensive, its persistent record may therefore approximate a detailed computational profile of a person's biological state, behavior, habits, and environment. This creates risks not only of unauthorized access, but also of continuous surveillance by healthcare providers, insurers, employers, family members, or other actors if access control is poorly defined.

Continuous surveillance can also affect autonomy and self-determination even when the data are collected with consent. A health digital twin is intended not merely to observe but often to predict future states and recommend interventions. For instance, a diabetes twin that detects declining activity and worsening glucose patterns might repeatedly recommend exercise, dietary changes, or medication adjustments; a cardiovascular twin might continuously warn an individual that particular behaviors increase predicted cardiac risk. Such recommendations may be clinically beneficial, but persistent monitoring and nudging can blur the boundary between assistance and behavioral control (März et al., 2025). Users may begin changing their behavior because they know they are continuously monitored, or may feel compelled to follow recommendations because the twin presents them as necessary for avoiding predicted adverse outcomes. In more consequential settings, recommendations could also influence treatment eligibility, insurance decisions, or clinicians' judgments, making it difficult for patients to disregard the computational representation of their future health.

Thus, surveillance in DT4H should not be treated simply as a stronger version of conventional health-data collection. The defining features of digital twins, including persistent observation, multimodal data integration, inference of latent characteristics, longitudinal prediction, and feedback into patient behavior, create a qualitatively deeper, more intimate, and tightly coupled relationship between the individual and the computational system. Ethical frameworks for DT4H should therefore incorporate stronger requirements for data minimization, transparency regarding inferred information, and meaningful patient control over which data streams are incorporated into the twin and how predictions are used. This is particularly important because increasing the fidelity of a digital twin creates an inherent tension: the more completely the system attempts to represent a person, the more extensively that person must be observed.

**Dynamic, Granular and Revocable Consent:** Dynamic, granular, modular, and revocable consent should be treated as a central extension of traditional informed consent for AI4H. In conventional AI4H applications, consent is often obtained for a relatively bounded purpose, such as using a particular dataset for diagnosis, prediction, or research. DT4H systems, by contrast, may continuously integrate heterogeneous data, update the patient's digital representation over time, and reuse that representation for multiple purposes, including diagnosis, treatment, disease-progression prediction, preventive intervention, and research (Mulder et al., 2022; Kuersten, 2023). A one-time, broadly worded consent therefore may not remain meaningful as the twin evolves. Dynamic consent addresses this problem by allowing consent decisions to be revisited as data sources, purposes, recipients, or system capabilities change. More generally, dynamic consent has been proposed as an ongoing, digitally mediated relationship in which individuals can review and modify consent preferences over time rather than making a single irreversible decision at enrollment (Kaye et al., 2015).

For DT4H, such consent should also be granular and modular. Granular and modular consent allows patients to make separate choices about different categories of data, uses, and recipients. For example, a patient with a cardiovascular digital twin might consent to continuous heart-rate and blood-pressure data being used for clinical monitoring, but decline the incorporation of location data, social-media activity, or behavioral information for lifestyle inference. The same patient might permit the twin to be used by their cardiologist for treatment planning while withholding permission for use by commercial partners or for unrelated research. Such flexibility is particularly important because DT4H can draw on data far beyond conventional medical records, and the scope of data collection may not be fully apparent to patients at the outset (Huang et al., 2022). It may also generate new derived information whose sensitivity was not foreseeable when the original data were collected. Consequently, meaningful consent must be an ongoing process. Individuals should consent not only to the initial data collection but also to any substantial changes, such as new downstream applications, the integration of new data streams, or access by third parties.

Consent must furthermore be revocable. Patients should be able to withdraw permission for particular data streams or uses without necessarily having to abandon the entire digital twin. For instance, a patient might initially allow continuous sleep data from a wearable to update the twin but later decide that this monitoring is too intrusive; revocable consent would allow that stream to be discontinued while preserving other clinically necessary components of the twin. This requirement is especially important because medical digital twins can involve repeated secondary uses of physiological data across treatment, research, and potentially commercial contexts. Shao and Ou (2025) explicitly argue that one-time broad consent is poorly suited to such repeated reuse and propose dynamic consent in which individuals are informed when the application context changes and can terminate consent over time.

**At the same time, dynamic and granular consent creates a practical tension:** Repeatedly asking patients to approve every minor update or data transaction could produce consent fatigue, cognitive burden, and superficial clicking rather than meaningful autonomy. Dynamic consent should therefore not mean continual interruption. Instead, DT4H governance should support configurable consent preferences, clear notification of material changes, accessible records of current permissions and past uses, and opportunities to revise or withdraw those permissions at meaningful decision points. The objective is to preserve continuing patient agency while avoiding a consent process so burdensome that meaningful control becomes impractical.

**Model Governance and Accountability:** Health digital twins are intended to provide dynamic, individualized representations capable of simulating patient-specific trajectories and interventions, often by integrating information dynamically across multiple biological and clinical scales (Vallée, 2026; Sel et al., 2025). Because human physiology is complex, heterogeneous, and continuously evolving, maintaining an accurate digital representation may require repeated updating, calibration, simulation, and validation (Menon et al., 2025).

These characteristics require governance to move beyond conventional model-level validation toward patient-level representational governance. A conventional clinical AI system may achieve acceptable aggregate performance on a validation population while still performing poorly for particular individuals. For a digital twin, this is a more fundamental concern because the system is intended to represent and simulate the state and trajectory of a specific patient. Sel et al. (2025) therefore emphasize the importance of Verification, Validation, and Uncertainty Quantification (VVUQ) for personalized validation methodologies and metrics rather than relying solely on conventional population-level evaluation. For example, a cardiovascular twin becomes less representative after the patient develops renal disease, changes medication, or becomes less physically active. The underlying physiological model may remain scientifically valid, while the instantiated twin no longer faithfully represents that individual. DT4H governance should therefore establish procedures for continuous verification and validation, uncertainty quantification, divergence monitoring, when a twin is no longer sufficiently reliable for its intended clinical use (Sel et al., 2025; Vallée, 2026).

DT4H also requires an extension from conventional model governance to simulation governance. Digital twins may be used not merely to classify or predict an observed condition, but to simulate alternative futures and patient-specific interventions (Iqbal et al., 2025; Vallée, 2023). For example, an oncology twin might compare several treatment regimens and estimate how a particular patient's tumor would respond to each. Such counterfactual outcomes have not actually occurred and may depend on incomplete biological knowledge, modeling assumptions, estimated parameters, and uncertain patient-specific data. Validation must therefore address not only predictive accuracy but also the fidelity of counterfactual and treatment-response simulations (Vallée, 2026; Sel et al., 2025). Governance frameworks should consequently require provenance and uncertainty reporting that clearly distinguish observed patient data, inferred physiological states, predicted trajectories, and simulated counterfactual outcomes. This distinction is particularly important when simulation outputs directly inform high-stakes clinical decisions.

The distributed and adaptive nature of DT4H also creates distinctive safety, accountability and liability challenges. A digital twin may combine data from sensors and electronic health records, simulation engines, clinical workflows, and technologies supplied by different organizations. Suppose a hospital uses a proprietary oncology twin to recommend treatment. The patient's clinical data come from the hospital, a vendor supplies the simulation engine, a third party supplies genomic analysis, and a clinician selects a treatment based on the simulated outcome. If harm occurs, who should be held responsible? Recent ethical and legal reviews identify liability attribution as an important unresolved issue because a harmful DT-supported decision may emerge from interactions among software developers, healthcare professionals, institutions, data providers, and continuously evolving computational components rather than from a single identifiable failure (Huang et al., 2022). Recent reviews similarly characterize healthcare-DT governance and liability frameworks as fragmented and poorly suited to complex, continuously evolving virtual patient representations. DT4H governance therefore requires stronger mechanisms for end-to-end provenance, version control, auditability, and distributed accountability, so that investigators can reconstruct which data, models, assumptions, simulations, devices, and human decisions contributed to a particular clinical outcome.

Overall, DT4H extends conventional AI4H model governance to ensure not only that an underlying model remains technically valid, but also that the instantiated twin continues to faithfully represent the particular patient; that changes to the twin are controlled and traceable; that simulations and their uncertainties are appropriately validated and communicated; and that interactions between computational outputs and real-world interventions remain safe over time. This broader governance perspective is consistent with emerging calls for digital-twin-specific validation, regulatory adaptation, and lifecycle oversight (Sel et al., 2025; Iqbal et al., 2025; Vallée, 2026).

**Bias and Fairness:** Bias and fairness concerns in DT4H build directly on longstanding concerns in AI4H but become more complex because a digital twin is intended to provide a persistent, patient-specific representation that evolves over time. A key extension is therefore to move from population-level fairness to patient-level representational fairness. A conventional AI4H model may be considered reasonably fair if performance disparities across demographic groups remain within acceptable bounds. For DT4H, however, group-level performance is not sufficient because the system claims to represent a particular individual. Fairness evaluation should therefore ask not only whether average model performance is comparable across populations, but also whether the fidelity, uncertainty, and clinical usefulness of patient-specific twins are equitable across groups.

A second DT4H-specific concern is bias accumulation and propagation across data modalities and over time. A digital twin may combine genomic data, imaging, laboratory measurements, wearable sensors, behavioral data, and environmental information. Each source can contain a different form of bias, and those biases can interact when integrated into the twin. Because DT4H systems evolve, bias can also become longitudinal and self-reinforcing. Consider a diabetes twin that initially has sparse information about a patient's diet and activity because the patient lacks reliable wearable connectivity. The twin may produce less accurate predictions, leading to less precisely tailored recommendations. If future model updates rely partly on the outcomes generated under those recommendations, initial representational deficiencies may persist or compound. Similarly, if a twin systematically underestimates disease risk for an underrepresented population, fewer preventive interventions may be recommended for those patients; subsequent clinical records may then reflect reduced diagnosis or treatment, creating new data that appear to validate the original biased assumptions. This type of feedback means that fairness cannot be assessed only during model development. DT4H requires continuous fairness auditing across the lifecycle, including monitoring of representation quality, prediction error, uncertainty, recommended interventions, and clinical outcomes across relevant patient groups. Vallée (2026) explicitly identifies bias propagation and unequal prediction/intervention quality as main challenges for digital twins.

A third extension concerns fairness of simulations and counterfactual recommendations. Conventional AI fairness typically evaluates whether predictions or classifications differ unjustifiably across groups. Digital twins may go further by simulating alternative treatments and recommending the intervention predicted to be best for an individual. Fairness must therefore extend to the quality of these counterfactual simulations.

A fourth concern is that equitable access becomes part of algorithmic fairness itself. High-fidelity DT4H may require continuous biomonitoring, genomic or multi-omic testing, advanced imaging, reliable digital connectivity, and healthcare infrastructure capable of maintaining and interpreting a twin. If such resources are disproportionately available to affluent patients or well-resourced healthcare systems, advantaged groups may receive increasingly accurate and individualized twins while disadvantaged populations receive lower-fidelity versions or no twin at all. Reviews of healthcare digital twins already identify fairness and socioeconomic disparities as persistent concerns (Ringeval et al., 2025). This creates a potential digital-twin divide. Therefore, DT4H fairness cannot be addressed solely by balancing training datasets. It must also consider equitable access to sensing technologies, data-generating infrastructure, computational resources, and clinical expertise needed to benefit from the twin.

**Ownership and Access Control:** Ownership and access-control concerns in DT4H extend beyond those typically encountered in conventional AI4H because the object being governed is no longer only a dataset or model output, but a persistent and evolving digital representation of a particular person. In AI4H, ownership questions commonly concern who controls patient data, who owns an AI model, and who may use the resulting predictions. A health digital twin complicates this structure because it may combine patient-generated data, EHRs, genomic information, wearable and sensor data, proprietary algorithms, inferred physiological states, and counterfactual simulation outputs within a single evolving system. Reviews of healthcare digital twins explicitly identify data ownership and governance as unresolved ethical and implementation challenges (Huang et al., 2022; Vallée, 2024). The resulting twin may therefore have no obvious single “owner”: the patient is the subject and principal source of much of the information, a hospital may operate the system and contribute clinical data, a technology company may own the modeling software, and other vendors may supply wearable devices or specialized simulation modules.

For this reason, DT4H governance should move beyond a simple notion of ownership toward clearly specified rights and responsibilities, including who may access, query, modify, simulate, transfer, commercialize, archive, or delete the twin. This distinction is important because ownership of one component should not automatically imply unrestricted control over the entire digital representation. For example, a company may own the software used to construct a cardiovascular twin, but this should not necessarily give it the right to use the patient's twin to develop unrelated commercial products or to provide access to insurers. Likewise, a hospital may have legitimate access to the twin for clinical care, while a research group may require separate authorization to run new simulations on the same representation. Huang et al. (2022) emphasize that digital twins involve multiple stages of data collection, management, analysis, and use, each of which introduces distinct questions about access, privacy, consent, and secondary use. DT4H therefore requires purpose-specific and role-based access governance, rather than treating access to the twin as a single binary permission.

Finally, ownership and access become particularly distinctive because a sufficiently persistent digital twin may outlive the patient. Conventional clinical records already raise post-mortem privacy questions, but a digital twin may remain an active computational representation capable of generating new simulations after death. De Kerckhove (2021) raised early ethical questions concerning the status and control of personal digital twins, and more recent work specifically identifies substantial governance gaps around post-humous digital twins. Cunneen et al. (2026) note that existing legal frameworks can leave uncertain who may continue, erase, monetize, donate, or otherwise use such digital representations after death, and propose advance data directives and stronger post-humous privacy and legacy rights. For example, could a pharmaceutical company continue running treatment simulations on a deceased patient's high-fidelity twin for drug research? Could family members authorize such use, or should the patient's prior wishes control? Should a twin be automatically archived or deleted at death? These questions make clear that DT4H requires lifecycle ownership and access governance, including rules for creation, use, modification, transfer, succession, archival, and deletion.

**Regulatory Compliance:** Conventional biomedical and AI4H regulatory frameworks provide an important foundation for DT4H, but their application should be extended to address the distinctive characteristics of persistent, evolving, and patient-specific digital twins. Recent reviews similarly emphasize that DT4H should be governed as a complex sociotechnical system rather than as an isolated algorithm or medical device (Katsoulakis et al., 2024; Burr et al., 2026). Building on these foundations, we identify several principles for adapting existing legal and regulatory approaches to DT4H.

First, *lifecycle and continuous governance* should extend oversight beyond discrete stages of data collection, model development, or deployment. Because DT4H continuously integrates heterogeneous data, updates patient-specific representations, and may operate across clinical, research, consumer, and commercial contexts, governance should consider the twin throughout its full lifecycle, including creation, active use, transfer, archival, termination, and, where relevant, post-humous use. Existing data-protection and consent principles should therefore extend to raw, derived, inferred, and simulated information, while preserving meaningful and revisable patient control as data sources, purposes, recipients, and system capabilities change over time.

Second, *patient-specific validation and regulatory assurance* should extend conventional episodic, population-level validation and approval toward continuous, patient-specific assessment throughout the DT4H lifecycle. Population-level validation alone is insufficient to determine whether an instantiated twin remains reliable, representative, and clinically appropriate for a specific patient as their condition and the twin evolve. DT4H therefore requires continuous patient-level *Verification, Validation, and Uncertainty Quantification (VVUQ)*, including patient-specific recalibration, assessment of representational fidelity, traceable monitoring of system changes, and ongoing validation to ensure that the twin remains fit for its intended clinical use (Lal et al., 2022).

Third, *simulation governance* should extend regulatory attention beyond conventional predictions and classifications to patient-specific simulations and counterfactual outcomes. Because such outputs may depend on inferred physiological states, model assumptions, uncertain parameters, and hypothetical interventions, governance should emphasize simulation validity, uncertainty, provenance, and intended use. It should also distinguish clearly among observed patient data, inferred states, predicted trajectories, and simulated counterfactual outcomes, particularly when these outputs inform clinical decisions (Katsoulakis et al., 2024; Burr et al., 2026).

Fourth, *system-level coordinated governance* should reflect the interconnected and distributed nature of DT4H. A digital twin may integrate sensors, EHRs, cloud infrastructure, physiological models, AI components, simulation engines, and clinician-facing systems supplied by multiple organizations. Governance should therefore consider risks, responsibilities, and interactions across the entire data-model-simulation-device-clinician ecosystem rather than evaluating components in isolation. It should also promote technical and semantic interoperability, including compatible data formats, standardized data-fusion practices, common clinical ontologies, and consistent validation approaches, while coordinating data-protection, medical-device, AI, cybersecurity, and clinical governance frameworks across institutions and jurisdictions.

Fifth, *rights-based governance of digital representations* should recognize that DT4H can generate information and digital assets that extend beyond the original patient data, including inferred health states, predictive risk profiles, patient-specific model parameters, and simulation outputs. Relevant rights and safeguards should therefore extend to these derived representations. Legitimate intellectual-property and commercial interests should be balanced against the transparency needed to support patient autonomy, clinical safety, auditability, and accountability (Burr et al., 2026).

Together, these extensions shift the regulatory perspective from governing discrete datasets, algorithms, or medical-device functions toward governing the *full lifecycle of an evolving patient-specific digital representation*. Rather than replacing existing biomedical and AI4H frameworks, they provide principles for adapting those frameworks to the distinctive representational, adaptive, simulation-based, interconnected, and context-dependent characteristics of DT4H.

**Social Implications:** DT4Hs may also generate broader social consequences that extend beyond conventional AI4H concerns. For example, although DT4H is primarily intended to improve prevention, diagnosis, and treatment, its simulation capabilities may also be extended beyond therapeutic purposes toward enhancement of human capabilities, raising broader philosophical questions about the boundaries of medicine, human identity, and biological lifespan (Bruynseels et al., 2018).

We further elaborate the proposed DT4H framework by connecting it with leading international bioethics and AI ethics frameworks, as summarized in Table 4. Our framework is not intended to replace established bioethical or AI ethics principles. Rather, it systematically integrates, extends, and adapts them to address the specific requirements that arise from the distinctive architecture, characteristics, and governance challenges of DT4H systems. Accordingly, the novelty of our framework lies not in introducing entirely new ethical principles, but in providing a conceptual bridge that translates established bioethical and AI4H governance principles into concrete ELSI requirements for DT4H.

**Table 4:** Comparison of leading international frameworks with the proposed ELSI DT4H framework.

Framework	Description	General principles	DT4H extensions
Classic bioethical principles	Establishes general guidelines for human-subjects research and healthcare	Autonomy, justice, non-maleficence, beneficence and utility	Foundations of the proposed ELSI framework for DT4H
WHO ethics and governance for AI4H	Extends general ethical AI principles to health-specific settings	Human autonomy, wellbeing and safety, transparency, responsibility, inclusiveness, equity, sustainability	Dynamic, granular, and revocable consent, patient-level representational governance and fairness, simulation governance and counterfactual validation, hypercollection and continuous surveillance, continuous lifecycle regulation and post-humous autonomy and legacy rights
EU AI Act	Establishes AI regulation and governance for EU	Risk management, privacy, data quality, transparency, human oversight, accountability, robustness and cybersecurity	Cross-jurisdictional coordination, simulation governance and counterfactual transparency, continuous human oversight and operational autonomy, distributed regulatory accountability, continuous calibration, accuracy, and cybersecurity
NIST AI RMF	Establishes an AI risk management framework	Reliability, safety, security, accountability, transparency, explainability, privacy, fairness	Lifecycle governance and continuous revalidation, patient-level representational fairness, dynamic, granular, and revocable consent, hypercollection and continuous surveillance, simulation governance and counterfactual provenance, distributed accountability and provenance tracking, post-humous data governance, digital-twin divide
FUTURE-AI	Establishes a healthcare-specific trustworthy AI framework	Fairness, universality, traceability, usability, robustness, explainability	Patient-level representational fairness, longitudinal bias propagation, end-to-end continuous provenance, dynamic, granular, and revocable consent, simulation and counterfactual validation, surveillance minimization and autonomy, digital-twin divide

### 4.3 ELSI considerations across the DT4H lifecycle

Because ELSI issues are deeply interconnected throughout the DT4H lifecycle, we next organize these concerns according to the key stages of the DT4H workflow. Figure 5 organizes the ELSI concerns identified in this survey across four major stages of the DT4H workflow: data acquisition, digital twin modeling, simulation and prediction, and clinical decision support. At the *data acquisition* stage, the dominant concerns arise from the extensive collection and integration of multimodal patient data, including privacy and security, informed and dynamic consent, autonomy, transparency, bias and fairness, continuous surveillance, and ownership and access control. The *digital twin modeling* stage introduces concerns associated with both AI/ML and mechanistic modeling. Bias and fairness are particularly relevant to AI/ML models, while privacy and security also affect mechanistic modeling; shared concerns include transparency, model governance, and dynamic consent. At the *simulation and prediction* stage, concerns shift toward the responsible use of patient-specific predictions and counterfactual simulations, including autonomy, transparency, safety and accountability, bias and fairness, dynamic consent, model governance, and ownership and access control. Finally, the *user-interface and clinical decision-support* stage emphasizes privacy and security, autonomy, safety and accountability, dynamic consent, and ownership and access control, reflecting the direct interaction among DT outputs, clinicians, and patients.

**Figure 5:**
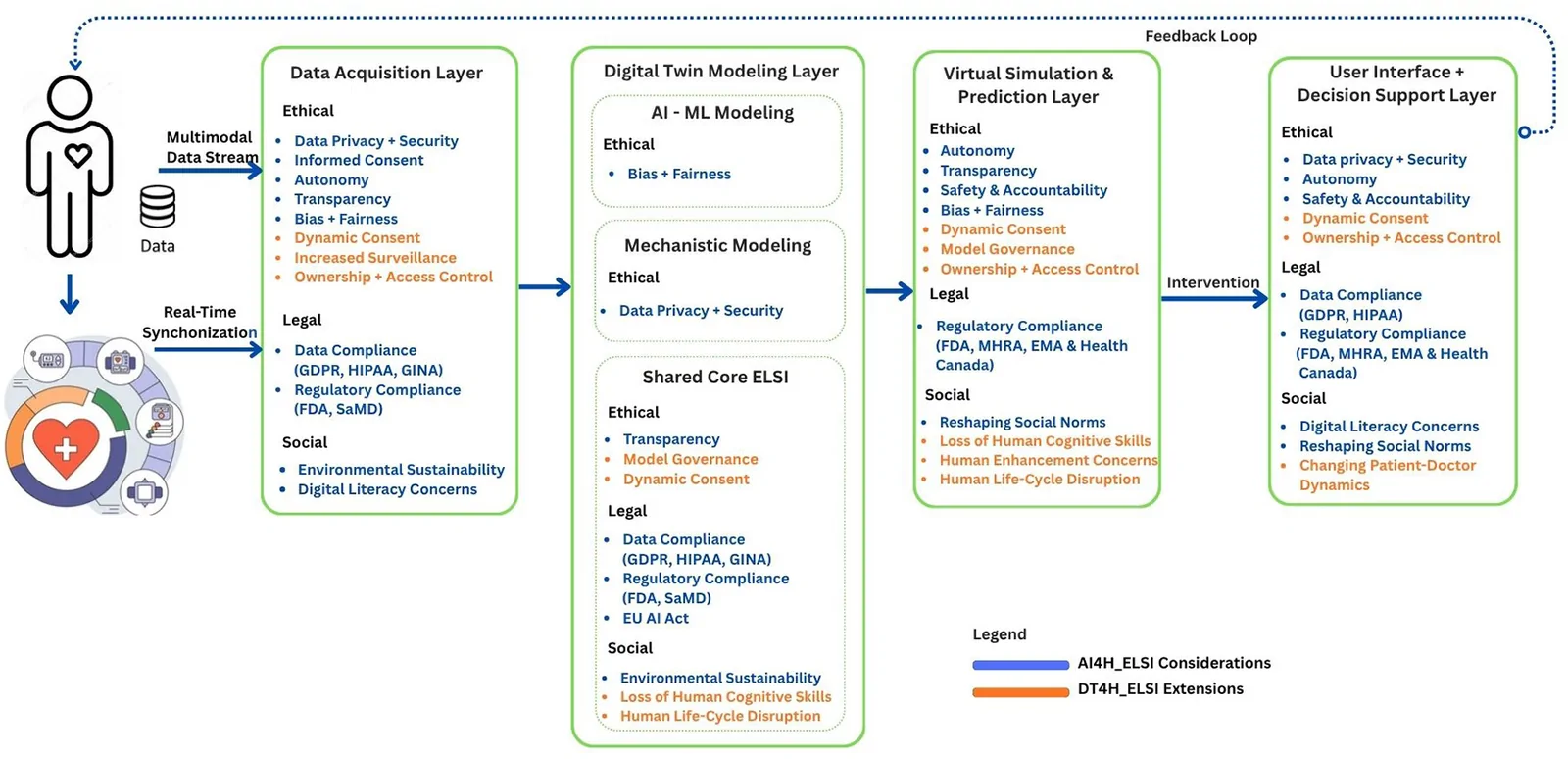
Illustrates ELSI implications in the DT4H workflow spanning data acquisition, digital twin modeling, simulation and prediction, and decision support.

Legal, regulatory, and societal considerations cut across these stages rather than belonging to a single point in the workflow. Data and regulatory compliance recur throughout the lifecycle, reflecting the need to govern continuously evolving DT4H systems rather than isolated data, models, or outputs. The figure also highlights broader societal concerns identified in the survey, including environmental sustainability, digital literacy, potential loss of human cognitive skills, changes in social norms and the patient-clinician relationship, human enhancement, and broader disruption of the human life cycle. Together, Figure 5 illustrates that DT4H ELSI concerns are interconnected and evolve as data move from data acquisition and modeling to simulation and ultimately to clinical decision-making.

## 5 Key legal and regulatory gaps

Our multi-layered ELSI framework for DT4H situates the key ELSI considerations of DT4H within established bioethical principles and ethical AI4H concerns. While the framework identifies the values, rights, and interests that should be protected, legal and regulatory frameworks provide mechanisms for translating many of these ethical requirements into enforceable obligations. It is therefore important to identify areas in which existing legal and compliance frameworks provide incomplete, uncertain, or insufficiently DT-specific protections. Addressing these gaps can provide a foundation for developing regulatory guidance that helps ensure that DT4H systems remain aligned with ethical principles throughout their lifecycle.

Based on the reviewed literature, legal and regulatory governance specifically addressing DT4H remains underdeveloped relative to the rapid development of these technologies. Existing health-data, medical-device, AI, anti-discrimination, liability, and intellectual-property frameworks provide important foundations but were generally not developed specifically for persistent, continuously evolving, patient-specific digital twins. Their application to DT4H therefore raises important coverage, interpretation, and implementation challenges. Our synthesis identifies several key gaps and limitations.

### 5.1 Data protection and privacy

Existing frameworks such as GDPR, HIPAA, and GINA provide important but different forms of protection and were not designed specifically for persistent, continuously evolving digital twins. HIPAA protects health information within specified covered entities and business associates, while GINA addresses certain uses of genetic information in health insurance and employment. However, DT4H may generate multimodal predictive profiles and derived biological information whose treatment under existing legal categories is not always clear, particularly when data originate outside conventional healthcare environments or predictions are derived from combinations of genomic, physiological, and behavioral data (Story, 2023). The reviewed literature also identifies concerns regarding unauthorized disclosure and subsequent misuse of DT data, including sensitive health information associated with stigmatized or legally contested forms of healthcare. Some studies further argue that existing remedies may be fragmented or limited when data breaches have not yet produced demonstrable downstream harm. In addition, anonymous and cross-jurisdictional attackers may be difficult to identify, attribute, prosecute, or hold civilly accountable following unauthorized access to DT systems (Story, 2023; Zoltick and Maisel, 2023).

Although GDPR is one of the most frequently cited privacy frameworks in the literature, applying some of its provisions to persistent digital twins may be complex. For example, GDPR provides a right to erasure, but this right is subject to exceptions, including circumstances involving legal retention requirements and specified public-interest purposes. Questions may therefore arise regarding how erasure, restriction, and retention should apply to continuously evolving DT data and derived representations. As noted by März et al. (2025), parents may consent to the use of their children's data in personalized DT systems, while those children may later seek to modify or withdraw consent after reaching adulthood. Some underlying health records may nevertheless remain subject to applicable legal or clinical retention requirements. This creates a potential tension between individual autonomy and institutional obligations to preserve health-related information. The literature also identifies weaknesses in cross-institutional mechanisms for detecting and preventing medical identity misuse and fraud (Teller, 2021).

Expanding on these privacy issues, many reviewed studies question whether conventional one-time informed consent is sufficient for DT4H systems. Dynamic consent is frequently proposed as an alternative, yet the literature provides limited detail regarding how it should be operationalized, when renewed authorization should be required, and how such mechanisms would interact with existing legal requirements. These questions are particularly important for populations with limited or evolving decision-making capacity, including minors, unconscious patients, and some older adults (März et al., 2025). Additional unresolved issues include how consent should be managed when a twin is created early in life, who may authorize initial and subsequent uses, what types of system changes should trigger renewed consent, and how withdrawal should affect continuing use of the twin or access to treatment (Braun, 2022).

### 5.2 Medical device and AI regulation

Existing medical-device and AI regulatory frameworks provide important foundations for DT4H but do not yet comprehensively address all characteristics of continuously evolving patient-specific twins. FDA and international regulatory efforts increasingly address AI-enabled medical devices, including lifecycle management, validation, post-market monitoring, and planned modifications. Nevertheless, the reviewed DT4H literature identifies limited DT-specific guidance concerning the validation and regulatory use of patient-specific simulations, *in silico* evidence, and standardized comparative evaluation of DT systems (Venkatesh et al., 2022). The literature also gives comparatively limited attention to the user-interface and user-experience requirements of healthcare professionals who must interpret DT predictions, simulations, and uncertainty estimates (Nelson, 2024).

Health Canada's Machine Learning-Enabled Medical Devices (MLMD) framework and the EU MDR provide risk-based regulatory foundations for software and AI-enabled medical devices, but important questions remain regarding their application to continuously updated, multimodal, and patient-specific DT systems (Lal et al., 2022). These include determining when patient-specific recalibration, incorporation of new sensors or models, or changes in simulation functionality require additional validation or regulatory review. In the United States, mechanisms such as Predetermined Change Control Plans provide a framework for prospectively specifying and validating certain planned modifications to AI-enabled device software. Comparable lifecycle principles may be useful for DT4H, although DT-specific requirements remain to be developed. In Europe, specific guidance has emerged for the clinical evaluation of orphan medical devices, but the EU does not provide an orphan-device pathway directly equivalent to U.S. mechanisms for rare-disease devices (März et al., 2025). Similarly, the EU AI Act establishes a risk-based framework that may apply to certain AI-enabled medical devices, but its interaction with the MDR and its application to continuously evolving DT4H systems require careful interpretation and coordination.

### 5.3 Anti-discrimination law

Existing anti-discrimination frameworks may provide only partial protection against some forms of discrimination enabled by DT4H. GINA protects against specified uses of genetic information in health insurance and employment, but its protections may not encompass all predictive biological profiles (Story, 2023). In addition, GINA does not extend its federal protections to life, disability, or long-term-care insurance, although some state laws provide additional protections. These limitations raise questions about whether computationally inferred disease risks and other DT-generated predictions and simulations should receive protections comparable to those afforded to legally defined genetic information. Existing laws may also provide fragmented protection against discriminatory or harmful use of leaked DT information associated with sensitive or legally contested forms of healthcare.

### 5.4 Liability and accountability

Another major concern raised in the literature is uncertainty surrounding liability and accountability. DT4H development and deployment may involve multiple stakeholders, including engineers, device manufacturers, software developers, healthcare institutions, clinicians, data providers, patients, and regulators. This distributed structure can complicate the application of existing malpractice, product-liability, and institutional-liability doctrines because harmful outcomes may arise from interactions among multiple components and actors rather than from a single identifiable failure.

Several studies describe a potential malpractice dilemma in which clinicians face uncertainty regarding how reliance on, or deviation from, DT-generated recommendations will be evaluated under evolving standards of care (Kuersten, 2023). Clinicians may be expected to exercise independent professional judgment while simultaneously being asked to incorporate increasingly complex computational recommendations whose assumptions, limitations, or uncertainty may not be fully transparent. Responsibility for adverse outcomes may therefore be difficult to allocate among clinicians, healthcare institutions, device manufacturers, software developers, and other system providers. Rather than assigning liability to a single actor, DT4H governance requires clearer allocation of responsibilities across the data-model-simulation-device-clinician chain, supported by end-to-end provenance, version control, audit trails, and documentation sufficient to reconstruct the decisions and system states contributing to an adverse outcome.

### 5.5 Intellectual property

Intellectual property, ownership, and control present additional challenges because a DT4H system may combine patient data, proprietary software, physiological models, patient-specific parameters, inferred states, and simulation outputs produced by different stakeholders. Existing intellectual-property, contract, privacy, and property-law doctrines may govern individual components, but there is no comprehensive legal framework that clearly allocates rights across the integrated and continuously evolving digital twin. This creates uncertainty regarding who may access, modify, transfer, commercialize, or reuse the twin and its derived outputs (Zoltick and Maisel, 2023).

Additional questions arise when DT4H systems generate potentially valuable scientific or technical outputs. Current U.S. patent and copyright frameworks generally require meaningful human inventorship or authorship, creating uncertainty regarding the ownership and protection of outputs produced through complex interactions between human contributors and AI-enabled DT systems. Governance must also address tensions between proprietary algorithms and trade secrets on the one hand and the transparency required for clinical safety, auditing, and accountability on the other.

### 5.6 Post-humous data governance

Post-humous governance presents another unresolved issue: who has authority over a patient's digital twin and associated biological and computational information after death? In the United States, there is no comprehensive federal framework specifically governing post-humous digital twins, although individual legal regimes may apply to particular components. Alaska law, for example, recognizes a DNA sample and the results of DNA analysis as the exclusive property of the individual from whom the sample was obtained, but this represents a specific statutory approach rather than a general U.S. rule governing digital twins. More broadly, U.S. law does not recognize a comprehensive property right in personal information comparable to ownership of tangible property, and existing frameworks provide limited DT-specific guidance regarding continued use of an evolving digital representation after death (Teller, 2021).

These limitations leave critical questions unresolved: What should happen to a digital twin after the patient dies? Can it remain computationally active or continue to incorporate population-level knowledge? Who may authorize its continued use, modification, research use, commercialization, archival, or deletion? Existing legal frameworks address some aspects of deceased persons' health information and research data, but no comprehensive framework specifically addresses the continued governance of persistent and potentially active DT4H representations (Amram et al., 2023).

### 5.7 Cross-jurisdictional harmonization and standardization

The reviewed literature identifies the absence of a unified international regulatory framework for DT4H as a significant governance gap. Although region-specific initiatives in the European Union, United States, China, Singapore, Hong Kong, and other jurisdictions provide important foundations, substantial challenges remain in regulatory harmonization, data governance, interoperability, standardized data-fusion processes, compatible clinical-data formats, and consistent system-validation practices (Qian et al., 2024). Further harmonization is also needed for clinical ontologies, biological parameter definitions, semantic representations, and modeling conventions to support reliable data exchange, model integration, and validation across DT4H systems and healthcare settings.

## 6 Main trade-offs in developing responsible DT4H

In practice, building an ethical, legally compliant, and clinically useful DT4H system requires navigating a series of difficult trade-offs. An ideal system would be accurate, transparent, privacy-preserving, fair, secure, fast, clinically useful, legally compliant, and cost-effective. However, these goals cannot always be optimized simultaneously. This tension is especially pronounced in DT4H because such systems depend on continuous multimodal data collection, real-time synchronization between physical and digital entities, adaptive model updating, and simulation-based prediction. Therefore, responsible DT4H development should not assume that a single optimal solution exists. Instead, it requires explicit, context-sensitive balancing of competing technical, ethical, legal, clinical, and economic priorities. In the following, we discuss several major trade-offs that may arise in developing ethical and responsible DT4H systems.

**Innovation vs. Regulation:** AI-driven DT4H technologies combine real-time multimodal data collection, predictive modeling and simulation, and clinical decision support. These capabilities create many opportunities for healthcare innovation, but they also challenge existing legal and regulatory frameworks, which often evolve more slowly than the technology itself. Inadequate DT4H oversight may undermine patient safety, human agency, and stakeholder accountability, whereas overly rigid regulation may create significant barriers to DT4H development and deployment (Coorey et al., 2022). This trade-off is particularly important because DT4H systems may be embedded in high-stakes clinical workflows. The absence of standardized data governance frameworks and clear regulatory pathways may discourage patients from sharing sensitive data and reduce stakeholder confidence in DT4H adoption. Unclear liability for adverse outcomes, such as unsafe treatment or drug dosage recommendations produced by unreliable model outputs, can further delay model development and clinical integration. Thus, DT4H governance must balance technological innovation with appropriate legal safeguards to support trustworthy and responsible adoption (Kuersten, 2023).

**Privacy vs. Utility:** DT4H systems require extensive personal health information, including genetic, physiological, behavioral, environmental, and lifestyle data, to construct high-fidelity digital replicas and support individualized prediction. In general, richer and more granular data can improve model accuracy, personalization, and clinical utility. However, the continuous collection and integration of such data increase risks of privacy loss, re-identification, cyber attacks, and unauthorized secondary use (Huang et al., 2022). For example, a heart DT requires a patient's live data stream, continuously integrating ECG records, wearable data, and biometric records. These high-resolution data can improve the accuracy of cardiac event simulations; however, continuous data collection could expose sensitive personal information. Therefore, de-identified datasets should be used to preserve the patient's privacy and reduce the risk of data breaches. Privacy-preserving techniques, such as data de-identification, and differential privacy, can reduce these risks (Menon et al., 2025). Yet these methods may also reduce data quality, remove clinically relevant signals, or introduce noise that limits downstream modeling and simulation. Developers must therefore balance privacy protection with the need to preserve sufficient utility for accurate and clinically meaningful DT4H functions.

**Accuracy vs. Fairness:** A central trade-off in ethical DT4H development is the balance between predictive accuracy and fairness. In conventional AI-based prediction, models are often optimized primarily to maximize overall accuracy or minimize prediction error. Fair AI methods modify this objective by adding fairness constraints or fairness-related penalty terms, or by formulating model training as a multi-objective optimization problem that jointly considers accuracy and fairness (Hardt et al., 2016; Islam et al., 2021). This means that improving fairness may require accepting some reduction in aggregate performance, especially when the available training data are imbalanced (e.g., less data on minorities). For example, ensuring that a DT4H model performs comparably well for minority or underrepresented groups may reduce overall population-level accuracy. However, in healthcare, maximizing average accuracy alone can be ethically problematic because poor performance for minority or marginalized groups may lead to unequal diagnosis, risk prediction, or treatment recommendations. Therefore, responsible DT4H development should evaluate both overall predictive performance and fairness, making explicit how accuracy-fairness trade-offs are defined, optimized, and justified (Barocas et al., 2023).

**Security vs. Responsiveness:** Because DT4H systems rely on sensitive biometric information, medical histories, behavioral data, and live data streams, they are natural targets for malicious attacks. Rigorous security measures, such as encryption, blockchain-based auditing, multi-factor authentication, intrusion detection, and strict access control, are necessary to protect patient data and system integrity. However, stronger security protocols may introduce computational overhead, slow data exchange, and reduce system responsiveness, especially in time-sensitive clinical situations (Jørgensen et al., 2023). This trade-off is especially relevant for DT4H systems that depend on real-time synchronization between the patient and the digital twin. Developers must therefore design security mechanisms that are strong enough to protect patients while remaining efficient enough to support timely clinical decision-making.

**Model Fidelity vs. Privacy:** AI-driven DT4H systems often require multimodal and multiscale data collected from live streams, including wearable sensors and clinical monitoring systems. Continuous synchronization can improve model fidelity, support more accurate prediction, and promote better health outcomes. However, pervasive monitoring may also diminish patient freedom and create heightened surveillance risks. In addition, DT4H data may reveal sensitive information not only about the patient but also about family members, partners, caregivers, or others who share the patient's environment. For example, a patient's heart rate, sleep patterns, cortisol levels, or location data collected through a smartwatch may indirectly reveal a partner's undisclosed habits or health-related behaviors. Thus, improving model fidelity through continuous monitoring may come at the cost of both individual and relational privacy (Teller, 2021). This trade-off highlights the need for data minimization, contextual consent, and safeguards against unintended disclosure of third-party information.

**Patient Effort vs. Model Performance:** Because DT4H systems are patient-centered and continuously updated, they often require active and sustained patient or caregiver engagement. Patients may need to wear sensors, provide self-reports, follow monitoring protocols, or interact with digital tools to maintain high-quality data streams. Such engagement can improve data completeness, model fidelity, and predictive performance. However, it may also impose burdens on patients and families, particularly in pediatric, chronic disease, disability, or rare disease contexts. Inconsistent participation can lead to missing data, model drift, unreliable predictions, and potential threats to patient safety (März et al., 2025). Developers must therefore balance the need for continuous high-quality data with usability, accessibility, patient comfort, and caregiver burden.

**Transparency vs. Model Performance:** DT4H systems often combine AI/ML models, mechanistic models, multimodal data fusion, and simulation engines. This technical complexity can improve predictive power and enable sophisticated “what-if” simulations, but it can also make the system difficult for clinicians, patients, and regulators to understand. Transparency is necessary for informed consent, clinical trust, accountability, and regulatory review. However, simpler and more interpretable models may not fully capture complex biological dynamics, while more powerful models may be less explainable. Developers must therefore balance technical sophistication with meaningful explainability. In DT4H, this may require different forms of explanation for different stakeholders, including patient-facing explanations of risks and choices, clinician-facing explanations of recommendations and uncertainty, and regulator-facing documentation of model assumptions, validation, and performance.

Overall, these trade-offs show that ethical DT4H development is not simply a matter of adding ELSI considerations after the system has been built. Instead, ethical, legal, social, technical, and clinical priorities must be integrated throughout the DT4H lifecycle. Developers should make trade-offs explicit, document the rationale behind design choices, engage patients and other stakeholders, and continuously reassess risks as DT4H systems evolve. Such an approach can help ensure that DT4H systems are not only innovative and clinically useful, but also safe, equitable, trustworthy, and socially responsible.

## 7 Recommendations

DT4H offers significant opportunities to improve individual wellbeing through precision medicine, more efficient clinical trials, and real-time clinical decision support. However, responsible implementation requires practical ethical and legal guidance that addresses ELSI concerns across the DT4H lifecycle. Based on the reviewed studies, we propose the following actionable guidelines for developing, deploying, and governing responsible DT4H systems.

**Dynamic, Granular, and Revocable Informed Consent:** DT4H systems should implement dynamic consent mechanisms that enable patients to review, manage, and revise their consent throughout the lifecycle of the digital twin. Developers, clinicians, healthcare institutions, compliance specialists, and end-users should jointly establish purpose-specific consent options, define which data streams are collected and at what frequency, specify when renewed consent is required, and document clear procedures for restricting or withdrawing consent. Consent decisions should be recorded in auditable, version-controlled logs so that current permissions, previous authorizations, and subsequent changes can be traced over time. Patients should also be clearly informed about what data are collected, how those data are used, how their digital twin is created and updated, who may access it, and how predictions and simulation outputs may influence clinical decisions.

These mechanisms should be implemented through accessible patient-facing interfaces that provide simple, granular controls for approving, restricting, modifying, or withdrawing permissions for specific data types, purposes, recipients, and secondary uses. Rather than requesting consent for every routine system update, DT4H systems should define explicit triggers for notification and re-consent when material changes occur. Such triggers may include the introduction of a new data source or sensor, a substantial change in model functionality, a new clinical or research purpose, the sharing of data with an additional organization or third party, or a change that materially affects the patient's risks or expected use of the twin. The system should notify patients of these changes in clear language, present the available choices and their consequences, and record the resulting decision. Where feasible, patients should also be able to withdraw consent selectively for particular data streams or uses without discontinuing the entire digital twin or unnecessarily disrupting clinical care.

**Ownership and Regulatory Compliance:** DT4H governance should clearly define rights and responsibilities over the evolving digital twin, including who may access, modify, simulate, transfer, commercialize, archive, or delete its different components. Access should be granular, purpose-specific, and role-based, and should extend beyond raw patient data to inferred states, simulation outputs, and other derived information. Clear contractual and intellectual property provisions should also specify rights over DT-generated outputs and biomedical insights, while remaining consistent with applicable privacy and genetic-information protections.

Regulatory compliance should be treated as a lifecycle responsibility. DT4H systems should be assessed under applicable medical-device and AI frameworks, including FDA guidance for SaMD, the EU MDR, Health Canada's MLMD framework, and the EU AI Act where relevant. Organizations should document intended use, validation evidence, and regulatory responsibilities, and reassess compliance when substantial changes occur after deployment. Cross-jurisdictional coordination through organizations such as the International Medical Device Regulators Forum (IMDRF) should also support greater harmonization in validation, interoperability, and safety monitoring.

**Data Privacy and Security:** Based on the findings of this survey, DT4H systems should establish privacy and security protections across the full data lifecycle, including data collection, integration, updating, inference, simulation, sharing, storage, and eventual archival or deletion. Protections should extend beyond raw health data to derived and simulated information, including inferred physiological states and predicted health trajectories. DT4H systems should comply with applicable privacy regulations, such as GDPR and HIPAA, and implement safeguards including encryption, multi-factor authentication, granular role- and purpose-based access control, regular security audits, and clear cross-border data-transfer policies. Where appropriate, privacy-preserving approaches such as federated learning should be considered to reduce unnecessary centralization of raw patient data. Particular attention should also be given to re-identification risk, especially when longitudinal genomic, physiological, behavioral, mobility, and wearable-device data are combined.

**Transparency and Explainability:** Developers should ensure that DT4H systems provide transparent and interpretable information about how digital-twin outputs are generated and updated. Systems should clearly distinguish observed patient data from inferred physiological states, predicted trajectories, and counterfactual simulation results, and provide traceable provenance for these outputs. Explanations should communicate the key factors influencing predictions or recommendations, together with relevant uncertainty and known limitations. Because DT4H models evolve over time, changes to the twin and their effects on outputs should also be documented and made understandable to clinicians to support appropriate interpretation and decision-making.

**Liability and Accountability:** Before deployment, DT4H stakeholders should explicitly define and document their respective responsibilities, including those of clinicians, developers, healthcare institutions, device manufacturers, software vendors, and patients. Liability arrangements should address not only failures attributable to a single actor or component, but also adverse outcomes arising from interactions among multiple stakeholders, data sources, devices, models, simulations, and clinical decisions. DT4H systems should therefore maintain end-to-end provenance and audit trails that capture data inputs, model and simulation updates, system recommendations, device status, and relevant human decisions. Clear human oversight, incident-reporting, and escalation procedures should also be established so that multi-component failures can be reconstructed, responsibilities can be identified, and corrective actions can be assigned.

**Continuous Surveillance and Patient Autonomy:** DT4H systems should preserve patient autonomy by limiting continuous monitoring to data necessary for clearly defined clinical purposes and allowing patients to control which data streams are collected and how observed and inferred information is used. Patients should be able to review, modify, or withdraw permissions as data sources, system capabilities, and intended uses change. DT4H interfaces should clearly communicate relevant predictions, recommendations, uncertainties, and uses of the twin, while preserving patient-clinician deliberation rather than replacing it. Organizations should also establish safeguards against excessive monitoring or undue influence from repeated DT recommendations and provide patients with clear mechanisms to question, decline, or discontinue non-essential DT-related monitoring or interventions where appropriate.

**Bias, Fairness, and Equity:** DT4H systems should address bias and equity throughout the lifecycle, from data collection and model development to validation, deployment, and continuous updating. Developers should identify underrepresented demographic and clinical groups in multimodal datasets, assess performance, uncertainty, and representational fidelity across these groups, and conduct periodic fairness audits to detect bias introduced or amplified as the twin evolves. Evaluation should extend beyond population-level accuracy to *patient-level representational fairness*, including whether individual twins provide comparable fidelity, uncertainty, and clinical usefulness across groups. Fairness assessments should also examine counterfactual simulations and treatment recommendations for systematic disparities.

To promote equity, healthcare institutions and technology providers should identify barriers to DT4H access, including unequal availability of sensing technologies, computational resources, and clinical expertise, and implement measures to reduce these gaps for underserved populations. Deployment decisions should consider whether certain groups are less likely to benefit from DT4H or more likely to experience lower-quality representations, and corrective actions should be taken before and after deployment when such disparities are identified.

**Stakeholder Collaboration:** DT4H projects should establish a multidisciplinary stakeholder group at the design stage and maintain engagement throughout development, validation, deployment, updating, and eventual retirement of the twin. Relevant stakeholders should include patients, caregivers, clinicians, biomedical engineers, AI developers, healthcare institutions, technology vendors, ethicists, legal experts, policymakers, and regulators. Organizations should assign clear roles and responsibilities and define when stakeholder input is required for decisions involving patient safety, autonomy, privacy, equity, model validity, and regulatory compliance. Patient input should be revisited when data sources, system capabilities, or intended uses materially change. Key decisions, stakeholder responsibilities, unresolved concerns, and subsequent changes should be documented throughout the DT4H lifecycle to support traceability, auditability, and accountability.

**Broader Social Implications:** DT4H deployment should include explicit safeguards against over-reliance on automated or simulation-based recommendations. Healthcare institutions should define when clinician review is required, preserve clinicians' authority to question or override DT outputs, and ensure that DT-supported decisions remain part of meaningful patient-clinician deliberation. Governance frameworks should also specify acceptable and prohibited uses of DT4H, particularly when simulation capabilities extend beyond prevention, diagnosis, and treatment toward human enhancement. Such uses should undergo additional ethical and institutional review to determine whether they are consistent with the intended goals and appropriate boundaries of healthcare.

**Digital Literacy Training:** DT4H stakeholders should provide accessible, role-specific digital literacy training for clinicians, patients, caregivers, and the broader public, with particular attention to underserved communities. Healthcare institutions, technology providers, government agencies, and community organizations should jointly develop training materials that explain DT4H capabilities, limitations, risks, and appropriate uses.

Clinicians should receive training on interpreting DT-generated predictions and simulations, communicating uncertainty, and integrating DT outputs into clinical judgment. Patients and caregivers should be taught how their data and digital twins are used and how to exercise their consent, access, modification, and withdrawal rights. Training should be offered through multiple formats, including in-person sessions, online modules, recorded videos, and written materials, and should be updated when DT4H systems, intended uses, or associated risks change. Organizations should also assess whether training materials are understandable and accessible to different user groups to support meaningful engagement and more equitable adoption.

## 8 Conclusions and future directions

In this survey, we analyze recent publications on ethical, legal, and responsible DT4H systems and synthesize the major ELSI themes in this emerging field. We ground the discussion in established bioethical principles and ELSI frameworks for AI4H systems to illustrate their interconnections and show how ELSI considerations for DT4H can be derived from, extended, and specialized beyond these existing frameworks. We also discuss the main trade-offs that arise when multiple objectives must be considered simultaneously in practice.

DT4H is still at an early stage, and both its technological capabilities and ELSI guidance continue to evolve. As next steps in ethical and responsible DT4H research, greater attention should be given to the following issues. First, stronger foundational ELSI guidelines are needed before DT4H systems are widely deployed. These guidelines should help standardize clinical practices, clarify medical terminology, and align ELSI documentation with relevant guidance from organizations such as the WHO and ISO standards. Future studies should focus on which ELSI categories are standardized across DT4H applications and which should remain context-specific based on the DT types, their usage, and patient demographics. Assessment should be conducted on whether this standardization improves clinicians' understanding of DT recommendations and supports more effective clinical decision-making.

Second, future ELSI frameworks should place stronger emphasis on equitable access to this new technology. DT4H systems should not benefit only well-resourced institutions or populations with greater access to digital technologies. Collaboration among public institutions, private-sector partners, healthcare providers, and community stakeholders is needed to support inclusive data collection, representative model development, and equitable access to DT4H-enabled care. Future research should examine the minimum infrastructure requirements such as EHR interoperability, availability of healthcare resources, and connectivity needs to develop clinically reliable DT systems.

Third, adopting dynamic, granular and revocable consent mechanisms should be considered a prerequisite for the data collection process in future DT4H project roadmaps. Studies should emphasize how ongoing consent can be implemented effectively, remain usable for patients who are cognitively impaired or older adults, and improve patient autonomy. Research should further investigate what security measures are needed for proxy consent obtained from family members, caregivers or legally appointed representatives which may introduce secondary ethical implications in sensitive healthcare environments.

Fourth, adaptive and integrated legal frameworks must evolve alongside DT4H systems. Because DT4H crosses boundaries among healthcare, AI, medical devices, data governance, and patient rights, future guidelines should be developed through multidisciplinary and cross-jurisdictional collaboration. Research should clarify how liability should be allocated when DT4H systems generate incorrect recommendations, clinicians rely on outdated DT recommendations, patients do not follow appropriate recommendations, or DT models change after deployment. Legal oversight, ethical governance, and clinical accountability should advance together with technological innovation to ensure that DT4H systems are safe, trustworthy, equitable, and socially responsible.

Finally, one limitation of this study is that our review was restricted to English-language publications, which may have excluded relevant DT4H ELSI research published in other languages and may introduce language bias into our synthesis. Consequently, the findings may not fully capture ethical, legal, and social concerns arising in different linguistic, cultural, and regulatory contexts. Future research should extend the review to non-English publications to provide a more comprehensive and globally representative understanding of ELSI in DT4H.

## References

[B1] Abrahams M. (2025). Digital twins: the future of personalized nutrition and health? Lifestyle Genomics 18, 59–63. doi: 10.1159/000543483 39947146

[B2] Acevedo J. C. Balazy P. James L. E. Nance D. N. (2026). “Digital twins in healthcare: creating a framework for ethical use,” in 2026 Systems and information engineering design symposium (SIEDS) (Charlottesville, VA: IEEE), 1–6. doi: 10.1109/SIEDS69358.2026.11540317

[B3] Alimour S. A. Alrabeei M. (2024). “A novel model for digital twins in mental health: the biopsychosocial AI-driven digital twin (badt) framework,” in 2024 11th International conference on software defined systems (SDS) (Gran Canaria: IEEE), 6–10. doi: 10.1109/SDS64317.2024.10883917

[B4] Amram B. Klempner U. Leibler Y. Greenbaum D. (2023). In their own image: ethical implications of the rise of digital twins/clones/simulacra in healthcare. Am. J. Bioethics 23, 79–81. doi: 10.1080/15265161.2023.2237456 37647469

[B5] Antoun I. Alkhayer A. Abdelrazik A. Eldesouky M. Thu K. M. Ibrahim M. . (2026). From polygenic risk to digital twins: the future of personalised cardiovascular medicine. Front. Cardiovasc. Med. 13:1735094. doi: 10.3389/fcvm.2026.1735094 41736831 PMC12926492

[B6] Armeni P. Polat I. De Rossi L. M. Diaferia L. Meregalli S. Gatti A. (2022). Digital twins in healthcare: is it the beginning of a new era of evidence-based medicine? A critical review. J. Pers. Med. 12:1255. doi: 10.3390/jpm12081255 36013204 PMC9410074

[B7] Attaran S. Attaran M. (2024). Advancing healthcare through the integration of digital twins technology: personalized medicine's next frontier. Fut. Internet 16:477. doi: 10.3390/fi16120477

[B8] Barbier M. Séroussi B. Bounaud-Devillers G. Desaive T. Duvignaud F. Elhadj E. . (2025). Digital Twins in Health Issues, Definitions and Ethical Challenges (Ph. D. thesis). Inria siège: Digital Health Agency (ANS).

[B9] Barocas S. Hardt M. Narayanan A. (2023). Fairness and Machine Learning: Limitations and Opportunities. Cambridge, MA: MIT Press.

[B10] Beauchamp T. L. (2008). “The Belmont report,” in The Oxford Textbook of Clinical Research Ethics, eds. E. J. Emanuel, C. C. Grady, R. A. Crouch, R. K. Lie, F. G. Miller, and D. D. Wendler (New York, NY: Oxford University Press), 149–155. doi: 10.1093/oso/9780195168655.003.0015

[B11] Begishev I. Shutova A. Akhmetshin E. Denisovich V. Latypova E. Gilmanov R. (2024). “Digital twins in healthcare system: communication between society and law,” in 2024 communication strategies in digital society seminar (ComSDS) (Saint Petersburg: IEEE), 9–12. doi: 10.1109/ComSDS61892.2024.10502083

[B12] Boretti A. (2025). Designing inclusive digital twins: ethical and practical considerations for trans healthcare. J. Med. Ethics 51, 803–804. doi: 10.1136/jme-2025-111120 40850759

[B13] Borrelli M. Musch S. Kohn B. Mishra A. Paul S. Chaffer T. J. . (2025). EU AI Act, harnessing digital-twin technology: how the innovative AI legislation provides regulatory safeguards to support the synergy between digital twin technology and general purpose AI systems. Off. J. Eur. Union. 48. doi: 10.2139/ssrn.5357031

[B14] Brand D. Devlin S. (2025). “Ethical, legal, and technical considerations for the development of digital twins in healthcare,” in 2025 IEEE 9th forum on research and technologies for society and industry (RTSI) (Tunis: IEEE), 308–313. doi: 10.1109/RTSI64020.2025.11212607

[B15] Braun M. (2022). Ethics of digital twins: four challenges. J. Med. Ethics 48, 579–580. doi: 10.1136/medethics-2021-107675 34380743

[B16] Braun M. Krutzinna J. (2022). Digital twins and the ethics of health decision-making concerning children. Patterns 3:100469. doi: 10.1016/j.patter.2022.100469 35465232 PMC9023882

[B17] Bruynseels K. Santoni de Sio F. Van den Hoven J. (2018). Digital twins in health care: ethical implications of an emerging engineering paradigm. Front. Genet. 9:31. doi: 10.3389/fgene.2018.00031 29487613 PMC5816748

[B18] Burr C. D. Qian S. Winter P. Chico T. Rangel Smith C. Wagg D. . (2026). “Realising the digital twin: a thematic review and analysis of the ethical, legal, and social issues for digital twins in healthcare,” *AI Soc*. 41, 1–25. doi: 10.1007/s00146-025-02833-6 42254856 PMC13241403

[B19] Busch F. Adams L. C. Bressem K. K. (2023). Biomedical ethical aspects towards the implementation of artificial intelligence in medical education. Med. Sci. Educ. 33, 1007–1012. doi: 10.1007/s40670-023-01815-x 37546190 PMC10403458

[B20] Carbonaro A. Marfoglia A. Quaranta L. Mellone S. Lanubile F. (2025). Editorial: implementing digital twins in healthcare: pathways to person-centric solutions. Front. Dig. Health 7:1741466. doi: 10.3389/fdgth.2025.1741466 41409538 PMC12706666

[B21] Coorey G. Figtree G. A. Fletcher D. F. Snelson V. J. Vernon S. T. Winlaw D. . (2022). The health digital twin to tackle cardiovascular disease—a review of an emerging interdisciplinary field. NPJ Dig. Med. 5:126. doi: 10.1038/s41746-022-00640-7 36028526 PMC9418270

[B22] Council for International Organizations of Medical Sciences. (2016). International Ethical Guidelines for Health-Related Research Involving Humans: Prepared by the Council for International Organizations of Medical Sciences (CIOMS) in collaboration with the World Health Organization (WHO). Geneva: Council for International Organizations of Medical Sciences. 40523065

[B23] Cunneen M. AnandFinn R. Friel R. Tennent P. Brandt S. (2026). From bones to bytes: anticipating and addressing the governance challenges of human digital remains and posthumous digital human twins. AI Soc. 41, 2021–2040. doi: 10.1007/s00146-025-02514-4

[B24] De Kerckhove D. (2021). The personal digital twin, ethical considerations. Philos. Trans. R. Soc. A 379(2207):20200367. doi: 10.1098/rsta.2020.0367 34398660

[B25] De Maeyer C. Renzi B. (2026). The shadow and the self in digital twins in healthcare as an AI environment. AI Soc. 41, 4949–4955. doi: 10.1007/s00146-026-02960-8

[B26] Deka N. Raut A. Samantaray S. Bhushan P. (2026). “Unlocking prospects of digital twin in healthcare,” in 2026 International conference on emerging systems and intelligent computing (ESIC) (Bhubaneswar: IEEE), 491–495. doi: 10.1109/ESIC68176.2026.11495875

[B27] Delerm F. Pilottin A. (2024). Double edged tech: navigating the public health and legal challenges of digital twin technology. Eur. J. Public Health 34(Suppl. 3):ckae144.1510. doi: 10.1093/eurpub/ckae144.1510

[B28] Drummond D. Coulet A. (2022). Technical, ethical, legal, and societal challenges with digital twin systems for the management of chronic diseases in children and young people. J. Med. Internet Res. 24:e39698. doi: 10.2196/39698 36315239 PMC9664337

[B29] Elgammal Z. Albrijawi M. T. Alhajj R. (2025). Digital twins in healthcare: a review of AI-powered practical applications across health domains. J. Big Data 12, 1–28. doi: 10.1186/s40537-025-01280-w

[B30] European Commission (2019). Ethics Guidelines for Trustworthy AI. Brussels: Technical Report, European Commission.

[B31] Ferlito B. De Proost M. Segers S. (2024). Navigating the landscape of digital twins in medicine: a relational bioethical inquiry. Asian Bioethics Rev. 16, 471–481. doi: 10.1007/s41649-024-00280-x 39022372 PMC11250715

[B32] Guerrero Qui nones J. L. Puzio A. (2025). Digital twins for trans people in healthcare: queer, phenomenological and bioethical considerations. J. Med. Ethics 51, 795–802. doi: 10.1136/jme-2024-110403 40037807

[B33] Hardt M. Price E. Srebro N. (2016). Equality of opportunity in supervised learning. Adv. Neural Inf. Process. Syst. 29, 3323–3331.

[B34] Huang P.-h. Kim K.-h. Schermer M. (2022). Ethical issues of digital twins for personalized health care service: preliminary mapping study. J. Med. Internet Res. 24:e33081. doi: 10.2196/33081 35099399 PMC8844982

[B35] Ibrahim S. Salhab N. (2023). “Digital twins for healthcare and telecommunications applications: a survey,” in 2023 4th International conference on data analytics for business and industry (ICDABI) (Manama: IEEE), 106–113. doi: 10.1109/ICDABI60145.2023.10629348

[B36] Iliuṭă M.-E. Trentesaux D. Moisescu M.-A. Mitulescu T.-C. (2025). “Ethical challenges of digital twins in medicine - normative guideline for glaucoma management,” in 2025 29th International conference on system theory, control and computing (ICSTCC) (Cluj-Napoca: IEEE), 830–835. doi: 10.1109/ICSTCC66753.2025.11240415

[B37] Iqbal J. D. Krauthammer M. Biller-Andorno N. (2022). The use and ethics of digital twins in medicine. J. Law Med. Ethics 50, 583–596. doi: 10.1017/jme.2022.97 36398633

[B38] Iqbal J. D. Krauthammer M. Witt C. M. Biller-Andorno N. Christen M. (2025). A consensus statement on the use of digital twins in medicine. NPJ Dig. Med. 8:484. doi: 10.1038/s41746-025-01897-4 40721854 PMC12304465

[B39] Islam R. Pan S. Foulds J. R. (2021). “Can we obtain fairness for free?,” in Proceedings of the 2021 AAAI/ACM conference on AI, ethics, and society (New York, NY: Association for Computing Machinery), 586–596. doi: 10.1145/3461702.3462614

[B40] Jabin M. S. R. Yaroson E. V. Ilodibe A. Eldabi T. (2024). Ethical and quality of care-related challenges of digital health twins in older care settings: protocol for a scoping review. JMIR Res. Protoc. 13:e51153. doi: 10.2196/51153 38393771 PMC10924255

[B41] Jørgensen C. S. Shukla A. Katt B. (2023). “Digital twins in healthcare: security, privacy, trust and safety challenges,” in European symposium on research in computer security (Cham: Springer), 140–153. doi: 10.1007/978-3-031-54129-2_9

[B42] Judijanto L. (2026). Bibliometric analysis of digital twin technology. West Sci. Soc. Human. Stud. 4, 716–726. doi: 10.58812/wsshs.v4i05.2904

[B43] Kamel Boulos M. N. Zhang P. (2021). Digital twins: from personalised medicine to precision public health. J. Pers. Med. 11:745. doi: 10.3390/jpm11080745 34442389 PMC8401029

[B44] Karimian G. Petelos E. Evers S. M. (2022). The ethical issues of the application of artificial intelligence in healthcare: a systematic scoping review. AI Ethics 2, 539–551. doi: 10.1007/s43681-021-00131-7

[B45] Katsoulakis E. Wang Q. Wu H. Shahriyari L. Fletcher R. Liu J. . (2024). Digital twins for health: a scoping review. NPJ Dig. Med. 7:77. doi: 10.1038/s41746-024-01073-0 38519626 PMC10960047

[B46] Kaye J. Whitley E. A. Lund D. Morrison M. Teare H. Melham K. (2015). Dynamic consent: a patient interface for twenty-first century research networks. Eur. J. Hum. Genet. 23, 141–146. doi: 10.1038/ejhg.2014.71 24801761 PMC4130658

[B47] Khan M. I. Yasmeen T. Khan M. Hadi N. U. Asif M. Farooq M. . (2025). Integrating industry 4.0 for enhanced sustainability: pathways and prospects. Sustain. Prod. Consumpt. 54, 149–189. doi: 10.1016/j.spc.2024.12.012

[B48] Khoshfekr Rudsari H. Tseng B. Zhu H. Song L. Gu C. Roy A. . (2025). Digital twins in healthcare: a comprehensive review and future directions. Front. Dig. Health 7:1633539. doi: 10.3389/fdgth.2025.1633539 41341463 PMC12671388

[B49] Kiseleva A. Kotzinos D. De Hert P. (2022). Transparency of AI in healthcare as a multilayered system of accountabilities: between legal requirements and technical limitations. Front. Artif. Intell. 5:879603. doi: 10.3389/frai.2022.879603 35707765 PMC9189302

[B50] Kuersten A. (2023). Health digital twins, legal liability, and medical practice. Am. J. Bioethics 23, 66–69. doi: 10.1080/15265161.2023.2237462 37647488

[B51] Kumar R. Kumar G. Upadhyay H. K. Kumar S. Bhat T. A. Kumar M. (2025). “Federated and explainable ethical digital twin framework for smart hospitals: towards responsible and trustworthy AI in healthcare,” in 2025 2nd International conference on artificial intelligence for innovations in healthcare industries (ICAIIHI) (Raipur: IEEE), 1–7. doi: 10.1109/ICAIIHI67124.2025.11403823

[B52] Lal A. Dang J. Nabzdyk C. Gajic O. Herasevich V. (2022). Regulatory oversight and ethical concerns surrounding software as medical device (SaMD) and digital twin technology in healthcare. Ann. Transl. Med. 10:950. doi: 10.21037/atm-22-4203 36267783 PMC9577733

[B53] Lekadir K. Frangi A. F. Porras A. R. Glocker B. Cintas C. Langlotz C. P. . (2025). FUTURE-AI: international consensus guideline for trustworthy and deployable artificial intelligence in healthcare. BMJ 388:e081554. doi: 10.1136/bmj-2024-081554 39909534 PMC11795397

[B54] Leong W. Y. (2025). “Digital twins in cyber-medical systems: revolutionizing personalized healthcare,” in International conference on applied system innovation (ICASI 2025) (Tokyo: IET), vol. 2025, 627–633. doi: 10.1049/icp.2025.2635

[B55] Li X. Loscalzo J. Mahmud A. K. M. F. Aly D. M. Rzhetsky A. Zitnik M. . (2025). Digital twins as global learning health and disease models for preventive and personalized medicine. Genome Med. 17:11. doi: 10.1186/s13073-025-01435-7 39920778 PMC11806862

[B56] Lomotey R. K. Kumi S. Ray M. Deters R. (2024). Synthetic Data Digital Twins and Data Trusts Control for Privacy in Health Data Sharing. Porto: Association for Computing Machinery, 1–10. doi: 10.1145/3643650.3658605

[B57] März J. W. Baumgartner M. Blau N. Biller-Andorno N. (2025). Digital twins for children with rare diseases: an exploration of the legal and ethical issues. Ethics Inf. Technol. 27:44. doi: 10.1007/s10676-025-09848-z

[B58] Masciari E. Umair A. Ullah M. H. (2024). A systematic literature review on AI-based recommendation systems and their ethical considerations. IEEE Access 12, 121223–121241. doi: 10.1109/ACCESS.2024.3451054

[B59] Mbiazi D. Bhange M. Babaei M. Sheth I. Kenfack P. Kahou S. E. (2025). Survey on AI ethics: a socio-technical perspective. Comput. Intell. 41:e70149. doi: 10.1111/coin.70149

[B60] Menon G. Malave B. Mhaske M. Parjane S. Mhaismale H. (2025). Digital twin technologies in medicine: the innovations, barriers, and future directions. Intell. Hosp. 2:100043. doi: 10.1016/j.inhs.2025.100043

[B61] Milosevic Z. Van Schalkwyk P. (2023). “Towards responsible digital twins,” in International conference on enterprise design, operations, and computing (Cham: Springer), 123–138. doi: 10.1007/978-3-031-54712-6_8

[B62] Mishra P. Rongali S. K. Manusha Reddy A. Nandihal P. R P. Prakash K. J. (2025). “Digital twin models for personalized healthcare diagnosis, treatment, and prognosis,” in 2025 IEEE 1st International conference on recent trends in computing and smart mobility (RCSM) (Bhopal: IEEE), 1–6. doi: 10.1109/RCSM67767.2025.11507206

[B63] Mulder S. T. Omidvari A.-H. Rueten-Budde A. J. Huang P.-H. Kim K.-H. Bais B. . (2022). Dynamic digital twin: diagnosis, treatment, prediction, and prevention of disease during the life course. J. Med. Internet Res. 24:e35675. doi: 10.2196/35675 36103220 PMC9520391

[B64] Narigina M. Romanovs A. Bruzgiene R. (2024). “Digital twin technology in healthcare: a literature review,” in 2024 IEEE 11th workshop on advances in information, electronic and electrical engineering (AIEEE) (Valmiera: IEEE), 1–8. doi: 10.1109/AIEEE62837.2024.10586661

[B65] National Institute of Standards and Technology (2023). Artificial Intelligence Risk Management Framework (AI RMF 1.0). Technical Report NIST AI 100-1. Gaithersburg, MD: National Institute of Standards and Technology.

[B66] Nelson B. J. (2024). “Regulatory challenges around digital twin models in medical devices,” in AAPM 66th annual meeting and exhibition (Los Angeles, CA: AAPM), 1.

[B67] OECD (2024). AI Principles. Technical Report. Paris: OECD.

[B68] Olawade D. B. Almarzook S. Ogunbona M. A. Makanjuola B. D. Olawuyi O. F. Wada O. Z. (2026). Digital twin applications in healthcare for people living with disability. Int. J. Med. Inform. 212:106359. doi: 10.1016/j.ijmedinf.2026.106359 41722355

[B69] Pan R. Sun H. Chen X. Pedrielli G. Huang J. (2025). “Human digital twin, precision healthcare, health monitoring, anomaly detection, statistical and machine learning models,” in 2025 IEEE international conference on data mining workshops (ICDMW) (Washington, DC: IEEE), 1–6. doi: 10.1109/ICDMW69685.2025.00195

[B70] Pandurangan K. Padmavathy B. S C. Vidyaa Nancy S. J. Mary J. L. R. M. (2025). “Assessment of digital twins in healthcare and future industry 6.0,” in 2025 3rd International conference on sustainable computing and data communication systems (ICSCDS) (Erode: IEEE), 1754–1761. doi: 10.1109/ICSCDS65426.2025.11167048

[B71] Peters M. D. Marnie C. Tricco A. C. Pollock D. Munn Z. Alexander L. . (2020). Updated methodological guidance for the conduct of scoping reviews. JBI Evid. Synth. 18, 2119–2126. doi: 10.11124/JBIES-20-00167 33038124

[B72] Piechowiak M. Goch A. Panas E. Masiak J. Mikolajewski D. Rojek I. . (2026). From local to global perspective in AI-based digital twins in healthcare. Appl. Sci. 16:83. doi: 10.3390/app16010083

[B73] Pisirgen A. Hiziroglu O. A. (2025). “Applications of digital twin in precision medicine: a systematic review,” in 2025 International conference on new trends in computing sciences (ICTCS) (Amman: IEEE), 392–399. doi: 10.1109/ICTCS65341.2025.10989480

[B74] Popa E. O. Van Hilten M. Oosterkamp E. Bogaardt M.-J. (2021). The use of digital twins in healthcare: socio-ethical benefits and socio-ethical risks. Life Sci. Soc. Policy 17:6. doi: 10.1186/s40504-021-00113-x 34218818 PMC8256511

[B75] Qian Y. Siau K. L. Nah F. F. (2024). Societal impacts of artificial intelligence: ethical, legal, and governance issues. Soc. Impacts 3:100040. doi: 10.1016/j.socimp.2024.100040

[B76] Ringeval M. Etindele Sosso F. A. Cousineau M. Parè G. (2025). Advancing health care with digital twins: meta-review of applications and implementation challenges. J. Med. Internet Res. 27:e69544. doi: 10.2196/69544 39969978 PMC11888003

[B77] Samei E. (2025). The future of *in silico* trials and digital twins in medicine. PNAS Nexus 4:pgaf123. doi: 10.1093/pnasnexus/pgaf123 40313535 PMC12043051

[B78] Saratkar S. Y. Langote M. Kumar P. Gote P. Weerarathna I. N. Mishra G. V. (2025). Digital twin for personalized medicine development. Front. Dig. Health 7:1583466. doi: 10.3389/fdgth.2025.1583466 40851640 PMC12369496

[B79] Sel K. Hawkins-Daarud A. Chaudhuri A. Osman D. Bahai A. Paydarfar D. . (2025). Survey and perspective on verification, validation, and uncertainty quantification of digital twins for precision medicine. NPJ Dig. Med. 8:40. doi: 10.1038/s41746-025-01447-y 39825103 PMC11742391

[B80] Shao Y. Ou Y. (2025). Leviathan-like concerns and reflections on medical digital twin technology. AI Soc.y. 41, 1–12. doi: 10.1007/s00146-025-02510-8

[B81] Sharma N. Panwar A. Sugandh U. Jain V. Singh Kaswan K. (2024). “Digital twins in healthcare: applications, challenges, and future directions,” in 2024 4th International conference on advancement in electronics and communication engineering (AECE) (Ghaziabad: IEEE). doi: 10.1109/AECE62803.2024.10911658

[B82] Silva A. Vale N. (2025). Digital twins in personalized medicine: bridging innovation and clinical reality. J. Pers. Med. 15:503. doi: 10.3390/jpm15110503 41295204 PMC12653454

[B83] Story B. (2023). Digital twins in healthcare: Proactive regulation to prevent a “runaway train”. North Carolina J. Law Technol. 25:315.

[B84] Sun Y. Ran L. (2025). The ethical risks and regulations of medical digital twin technology. Acta Bioeth. 31, 337–346. doi: 10.4067/S1726-569X2025000200337

[B85] Tahir M. Shafique A. Abid N. (2026). “A theoretical framework for AI-agentic systems integrated with digital twins: autonomous optimization of healthcare project,” in 2026 International conference on frontiers of engineering and emerging technologies (FET) (Sakhir: IEEE), 1–6. doi: 10.1109/FET68771.2026.11601432

[B86] Teller M. (2021). Legal aspects related to digital twin. Philos. Trans. R. Soc. A 379:20210023. doi: 10.1098/rsta.2021.0023 34398645

[B87] Tretter M. (2021). Perspectives on digital twins and the (im) possibilities of control. J. Med. Ethics 47, 410–411. doi: 10.1136/medethics-2021-107460 34001527

[B88] Tricco A. C. Lillie E. Zarin W. O'Brien K. K. Colquhoun H. Levac D. . (2018). PRISMA extension for scoping reviews (PRISMA-ScR): checklist and explanation. Ann. Intern. Med. 169, 467–473. doi: 10.7326/M18-0850 30178033

[B89] Vallée A. (2023). Digital twin for healthcare systems. Front. Dig. Health 5:1253050. doi: 10.3389/fdgth.2023.1253050 37744683 PMC10513171

[B90] Vallée A. (2024). Envisioning the future of personalized medicine: role and realities of digital twins. J. Med. Internet Res. 26:e50204. doi: 10.2196/50204 38739913 PMC11130780

[B91] Vallée A. (2026). Multi-scale digital twins for personalized medicine. Front. Dig. Health 8:1753906. doi: 10.3389/fdgth.2026.1753906 41778010 PMC12950698

[B92] Vanin J. Hagar A. Glazier J. A. (2026). Who's afraid of synthetic data? Hybrid approaches to deliver medical digital twins. Inform. Med. Unlocked 61:101737. doi: 10.1016/j.imu.2026.101737 41930261 PMC13041779

[B93] Varkey B. (2021). Principles of clinical ethics and their application to practice. Med. Principl. Pract. 30, 17–28. doi: 10.1159/000509119 32498071 PMC7923912

[B94] Vasiliu-Feltes I. Mylrea M. Zhang C. Y. Wood T.-C. Thornley B. (2023). Impact of blockchain-digital twin technology on precision health, pharmaceutical industry, and life sciences: conference proceedings, conv2x 2023. Blockchain Healthc. Today 6, 10–30953. doi: 10.30953/bhty.v6.281 38187956 PMC10770801

[B95] Vasiliu-Feltes I. Zhang C. Y. Dennis S. Siegel E. Uribe D. (2025). Trust by design: enabling responsible precision health through blockchain-powered digital twins and trusted ai. Blockchain Healthc. Today 8. doi: 10.30953/bhty.v8.453 41640846 PMC12865783

[B96] Venkatesh K. P. Brito G. Kamel Boulos M. N. (2024). Health digital twins in life science and health care innovation. Annu. Rev. Pharmacol. Toxicol. 64, 159–170. doi: 10.1146/annurev-pharmtox-022123-022046 37562495

[B97] Venkatesh K. P. Raza M. M. Kvedar J. C. (2022). Health digital twins as tools for precision medicine: considerations for computation, implementation, and regulation. NPJ Dig. Med. 5:150. doi: 10.1038/s41746-022-00694-7 36138125 PMC9500019

[B98] Venkatraman S. Fahd K. Wang X. Parvin S. Minicz J. (2026). Introducing digital twin capability building in healthcare through AI powered projects: AI powered project strategies in capability building and transformation of digital healthcare. Assoc. Comput. Mach. 1–9. doi: 10.1145/3774816.3774834

[B99] Verhees F. G. Wiest I. C. Kather J. N. Kambeitz J. Mikolas P. (2026). Blocking the reflection: milestones and hurdles for digital twins in mental health. Pharmacopsychiatry 59, 117–125. doi: 10.1055/a-2816-2869 41856487 PMC13155854

[B100] Vithya V. Valarmathie P. (2024). “Real-time monitoring in healthcare: Addressing technical and ethical issues with digital twin technology,” in 2024 International conference on smart technologies for sustainable development goals (ICSTSDG) (Chennai: IEEE), 1–8. doi: 10.1109/ICSTSDG61998.2024.11026075

[B101] World Health Organization (2021). Ethics and Governance of Artificial Intelligence for Health: WHO Guidance. Technical Report. Geneva: World Health Organization.

[B102] World Medical Association (2013). World Medical Association declaration of Helsinki: ethical principles for medical research involving human subjects. JAMA 310, 2191–2194. doi: 10.1001/jama.2013.281053 24141714

[B103] Xames M. D. (2025). Data inaccessibility is stifling the digital twin implementation in health care. J. Med. Internet Res. 27:e76524. doi: 10.2196/76524 40460307 PMC12151448

[B104] Xu G. Li X. Chen Y. Duan Y. Wu S. Yu H. . (2026). A comprehensive survey of AI agents in healthcare. J. Biomed. Inform. 179:105045. doi: 10.1016/j.jbi.2026.105045 42009269 PMC13435129

[B105] Zhang K. Zhou H.-Y. Baptista-Hon D. T. Gao Y. Liu X. Oermann E. . (2024). Concepts and applications of digital twins in healthcare and medicine. Patterns 5:101028. doi: 10.1016/j.patter.2024.101028 39233690 PMC11368703

[B106] Zoltick M. M. Maisel J. B. (2023). “Societal impacts: legal, regulatory and ethical considerations for the digital twin,” in The Digital Twin, eds. N. Crespi, A. T. Drobot, and R. Minerva (Cham: Springer), 1167–1200. doi: 10.1007/978-3-031-21343-4_37

